# Magnetoplasmonic Nanostructures from Magnetite with Noble Metal Surface Modification and Their Antimicrobial Activity

**DOI:** 10.3390/ijms262412092

**Published:** 2025-12-16

**Authors:** Helmina Ardeleanu, Maria-Crinela Ardeleanu, Simona Dunca, Marian Grigoras, Gabriel Ababei, Daniela Pricop, Laura Ursu, Georgiana Bulai, Daniel Timpu, Nicoleta Lupu, Alin Ciobica, Mihaela Racuciu, Dorina Creanga

**Affiliations:** 1 Physics Department, Alexandru Ioan Cuza University, 11 Carol I, 700506 Iasi, Romania; ardeleanu_helmina@yahoo.com (H.A.); georgiana.bulai@uaic.ro (G.B.); 2 Biology Faculty, Alexandru Ioan Cuza University, 20A Carol I, 700505 Iasi, Romania; ardeleanumaria425@yahoo.com (M.-C.A.); sdunca@uaic.ro (S.D.); alin.ciobica@uaic.ro (A.C.); 3 National Institute of Research and Development for Technical Physics, 47 Mangeron Blvd., 700050 Iasi, Romania; mgrigoras@phys-iasi.ro (M.G.); gababei@phys-iasi.ro (G.A.); nicole@phys-iasi.ro (N.L.); 4 Laboratory of Astronomy and Astrophysics, Research Center with Integrated Techniques for Atmospheric Aerosol Investigation in Romania (RECENT AIR), Alexandru Ioan Cuza University, 11 Carol I, 700506 Iasi, Romania; daniela.a.pricop@gmail.com; 5 P. Poni Institute of Macromolecular Chemistry, 41A Grigore Ghica Voda Alley, 700487 Iasi, Romania; ursu.laura@icmpp.ro (L.U.); dtimpu@icmpp.ro (D.T.); 6 “Ioan Haulica” Institute, Apollonia University, Pacurari Street 11, 700511 Iasi, Romania; 7 Biomedical Research Group, “Olga Necrasov” Center, Romanian Academy, Iasi Branch, Teodor Codrescu 2, 700481 Iasi, Romania; 8 Academy of Romanian Scientists, Ilfov No. 3, 030167 Bucharest, Romania; 9 Environmental Sciences and Physics Department, Faculty of Sciences, Lucian Blaga University of Sibiu, 5–7 Dr. I. Ratiu Street, 550012 Sibiu, Romania

**Keywords:** superparamagnetism, localized surface plasmon resonance, nanocomposites, *S. aureus*, *E. coli*

## Abstract

Multifunctional nanomaterials have been extensively investigated in theranostics to enhance therapeutic specificity, biocompatibility, and responsiveness to external magnetic gradients. We synthesized magnetoplasmonic nanocomposites comprising magnetite nanoparticles modified with gold and silver. Magnetite was synthesized via chemical co-precipitation and stabilized in an aqueous medium using glucose, which also served as a reducing agent for Au^3+^ and Ag^+^ ions on the nanoparticle surface. Microstructural, magnetic, spectral, and optical characterizations confirmed the successful formation of nanocomposites with properties suitable for biomedical applications. Plasmonic behavior was evidenced by visible-range absorbance maxima at 398 nm (Ag) and 538 nm (Au), while Transmission Electron Microscopy (TEM) revealed mean diameters of 21 and 23 nm. Zeta potential values of +23 mV for magnetite–silver and −40 mV for magnetite–gold nanocomposite samples indicated good suspension stability. Antibacterial activity against Gram-positive and Gram-negative bacteria was evaluated using agar diffusion and by determining the minimum inhibitory (MIC) and bactericidal (MBC) concentrations. Silver-modified magnetite nanocomposites exhibited the most potent effects, with MIC values of 0.01 mg/mL for *Escherichia coli* (*E. coli*) and 0.02 mg/mL for *Staphylococcus aureus (S. aureus)*, and corresponding MBC values of 0.027 mg/mL and 0.055 mg/mL, respectively. These magnetoplasmonic nanostructures have significant potential for overcoming antibiotic resistance and enabling targeted therapeutic action through magnetic guidance.

## 1. Introduction

Magnetic nanoparticles have received increased attention from nanotechnology researchers due to their diverse, ongoing, challenging, technical, and biomedical applications. In most applications in medicine and biology, magnetic nanoparticles are in contact with aqueous environments in living organisms; therefore, in addition to the need to ensure adequate magnetic and microstructural properties, it is very important to guarantee stability properties at the interface of these environments [1]. The need to protect the surface of magnetic nanoparticles against the risk of oxidation upon contact with water molecules and dissolved oxygen in intra- and extracellular environments raises the issue of reactivity at the interface between the nanoparticles and the periproximal environment. The modification of the surface of magnetic nanoparticles is discussed in the context of their coating with organic or inorganic shells that allows for functionalization at the same time, that is, in particular, the attachment of ligands of therapeutic interest, for example, drug molecules that need to be targeted as precisely as possible to reduce side effects [2].

Given their potential applications in biomedical settings, it is essential to elucidate the mechanisms underlying the antibacterial properties and magnetoplasmonic performance of noble metal-coated magnetic nanostructures, as outlined. To give them biomedical relevance, magnetite-based nanocomposites with noble metals exhibit synergistic antibacterial and magnetoplasmonic functionalities. Silver nanoparticles exhibit the best-known antimicrobial activity and are the most effective against bacterial microorganisms.

First, they can interact with sulfolipids and sulfur-aminolipids in the cell membrane, as well as with thiol-containing bacterial membrane proteins, which can directly affect the integrity and permeability of the bacterial membrane, leading to cell death [3]. Second, they can interact with proteins and DNA inside the cells, preventing cell proliferation [4]. Moreover, there is a remarkable mechanism provided by the release of Ag+ ions (re-oxidized silver atoms), which generates reactive oxygen species (ROS), and these inhibit cellular functions, such as respiratory pathways, and DNA replication, which leads to cell apoptosis [3]. According to Mobed et al. (2021), the main mechanism of the antibacterial activity of gold nanoparticles is the disruption of metabolic pathways, such as the ATP production pathway; however, they can also damage DNA molecules, cell membranes, and the electron transfer chain, including through the production of ROS [5]. According to Cui et al. (2012), in the case of gold–magnetite nanocomposites, the antimicrobial action of gold seems to be less significant than that of iron oxide, which is not based on reactive oxygen species (ROS) formation [6]. Chatterjee et al. (2011) demonstrated that gold nanoparticles have no antimicrobial effects on the Gram-negative strains of *E. coli* [7], and according to Gudkov et al. (2021), the combined use of iron oxide nanoparticles and of gold nanoparticles does not reduce the growth of *E. coli* cultures but affects its cell division [8].

Gold particles are especially effective against Gram-positive bacteria, such as *S. aureus* [9], since they electrostatically adsorb to the cell surface, interact with the amino acid lysine in the bacterial membrane, and cause the formation of pores that irreparably affect the integrity, functions, and viability of the cells. Although gold possesses lower inherent antibacterial activity, it provides a chemically inert and biocompatible interface that supports ligand conjugation via thiol or amine groups, thereby enabling selective targeting and reducing systemic toxicity [10].

The main mechanism of antimicrobial activity of iron oxides consists of the production of ROS through iron ions that are released into the cellular environment and react with the hydrogen peroxide normally present in cells in certain concentrations [11]. From the magnetoplasmonic perspective, the Fe_3_O_4_ core ensures magnetic responsiveness for field-guided spatial localization and potential hyperthermic activation under alternating magnetic fields, whereas the Au or Ag shells generate localized surface plasmon resonance (LSPR), typically in the visible–near-infrared range (λ ≈ 520–600 nm), producing confined photothermal effects that potentiate the disruption of microbial membranes [12,13]. The biocompatibility of these nanocomposites is supported by the metabolic integration of iron via ferritin-related pathways, the cytocompatibility of gold, and the ability to tune silver ion release via shell geometry or functionalization [14]. Furthermore, when integrated within biopolymer-based matrices, these hybrid magnetoplasmonic systems exhibit improved stability, bioadhesion, and responsiveness to external stimuli, representing an advanced approach for targeted antimicrobial therapy and theranostic applications [15].

Coating with noble metals, such as gold and silver, prevents agglomeration, ensures control of the interface’s reactivity, and allows for the insertion of functional groups, not to mention the antimicrobial action of silver, an effect that is necessary in any intervention in a patient’s body. In addition, the presence of noble metal layers provides the magnetic nanoparticles with special optical–spectral properties due to the phenomenon of localized surface plasmon resonance, which is very useful for sensoristic applications. The combined magnetic and optical properties of magnetite coated with noble metals make the nanoparticles suitable for magnetic resonance imagistic with enhanced contrast and enhanced local heating in photothermal therapy [3].

The study of the preparation and stabilization of magnetoplasmonic nanocomposites in aqueous media is of concern to numerous research teams.

An interesting magnetoplasmonic system was realized by Salazar Sandoval et al. (2023) by associating the magnetite/gold core/shell nanoparticles and using a β-cyclodextrin nanosponge to ensure stability in an aqueous medium for applications in drug encapsulation and controlled release [16].

Xing et al. (2015) synthesized magnetite nanoparticles coated with gold, considering that gold, being an inert but biocompatible element, could be very useful for protecting magnetic nanoparticles, giving them versatility in surface modification and high catalytic characteristics [17].

Munshi et al. (2016) reported gold-coated magnetite nanoparticles application in H_2_O_2_ electrochemical sensors that are very useful for various biomedical, healthcare, and industrial applications [18].

Due to their high biocompatibility, gold-coated nanoparticles have been studied for use in biosensors designed for the recognition or detection of biological entities (polypeptides, DNA, and polysaccharides) or as bio-signal transducers [19].

Mehdipour et al. (2021) proved that gold-coated magnetic nanoparticles are effective for bioseparation as well as for biosensing with specific applications in biosensors, microRNA detection, and surface-enhanced Raman scattering [20].

The antibacterial and antifungal activity of gold–magnetite nanocomposites was reported by Keihan et al. (2017), who tested the nanoparticles against representative public health microorganisms, both Gram-positive and Gram-negative bacteria, as well as against pathogenic fungi [21].

An efficient sensor based on silver-coated magnetite nanoparticles has been proposed to improve the sensitivity in the detection of clinical targets because silver coating increases the refractive index sensitivity and quality factor of the sensors, allowing for the more accurate detection of analytes at low concentrations [22].

Nanocomposites obtained from the direct reduction of silver ions on magnetite nanoparticles coated with polydopamine exhibited catalytic activity for 4-nitrophenol, methyl orange, and methylene blue, which recommends them for cleaning industrial wastewater from toxic compounds [23].

By embedding silver in porous magnetite, Singh et al. (2014) prepared some nanocomposites with significant antibacterial activity against Gram-negative and Gram-positive bacteria (*Escherichia coli* and *Bacillus subtilis*, respectively) [24].

The remarkable antimicrobial activity of silver-coated magnetic nanoparticles obtained by Park et al. (2019) was evidenced against food-borne *Escherichia coli* and *Salmonella* species based on optical density assays, bioluminescent imaging, and colony-forming unit assessments [25].

Magnetite–silver core–shell nanoparticles have been shown to be more effective than magnetite or silver nanoparticles in inhibiting some strains of pathogenic bacteria, such as *Salmonella typhimurium*, *Pseudomonas aeruginosa*, and *Staphylococcus aureus* [26], according to the minimum bactericidal concentration test.

Sharaf et al. (2022) synthesized silver/magnetite nanocomposites using trisodium citrate to couple magnetite nanoparticles with silver ones, their good antibacterial activity against the pathogen *Escherichia coli* was demonstrated by the diffusimetric method and estimation of the minimum inhibitory concentration [27].

Pieretti et al. (2020) produced magnetite nanoparticles combined with silver nanoparticles in a hybrid nanostructure as a useful tool in experimental oncologic treatment [28]. This is a promising approach for targeted cancer treatment because the nanoparticle combination exhibited selective bioeffects in tumoral cells, proving to be non-cytotoxic against healthy cells.

In accordance with recent advancements in the field of bio-nanocomposites, the creation of multifunctional hybrid systems that integrate inorganic nanostructures with biopolymers has emerged as a pivotal strategy for achieving enhanced biomedical efficacy [29,30]. The integration of magnetoplasmonic nanocomposites with either natural or synthetic biopolymers, such as chitosan, alginate, gelatin, or hyaluronic acid, yields improved biocompatibility, bio-adhesion, and stability within physiological environments while maintaining magnetic and plasmonic responsiveness to external stimuli, including magnetic fields or light irradiation [31,32,33]. These hybrid materials serve as intelligent platforms for controlled drug delivery, antimicrobial coatings, wound healing, and tissue regeneration, leveraging the magnetic core for spatial targeting and the plasmonic shell for photothermal activation or biosensing [34,35].

Within this context, the present study offers fundamental insights into the synthesis, structural and magnetic characterization, and antibacterial assessment of Fe_3_O_4_/Au and Fe_3_O_4_/Ag nanocomposites, which are envisioned as active components for future biopolymer-based theranostic architectures.

We studied the synthesis of magnetite/silver and magnetite/gold hybrid nanostructures to design suitable tools for magnetically targeted agents with plasmonic characteristics and antimicrobial properties as an advanced route for theranostic purposes.

## 2. Results

### 2.1. The Results of UV–Vis Spectrophotometry Analysis

**Figure 1:**
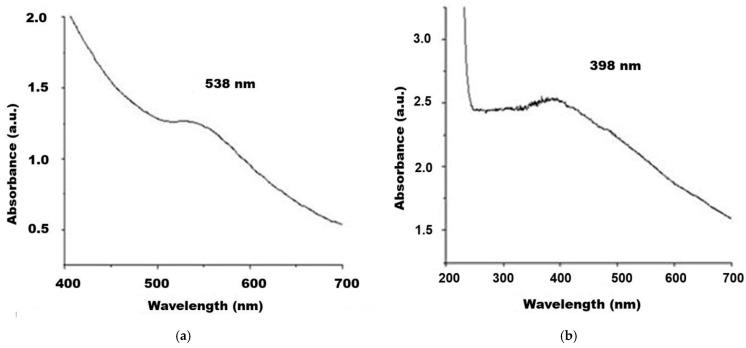
The UV–Vis absorption spectra of the nanocomposite samples prepared for characterization and tests: (**a**) Fe_3_O_4_@Au; (**b**) Fe_3_O_4_@Ag.

The first investigation method we applied was based on UV–Vis spectroscopy, which provides the direct identification of noble metal presence in the studied magnetoplasmonic nanostructures while also allowing for rapid monitoring of the synthesis’s progress. The spectra recorded in the UV–Vis range after graduate formation of the expected magnetoplasmonic hybrid nanocomposites are presented in Figure 1a,b.

For the Fe_3_O_4_@Ag sample we recorded the absorbance maximum at 398 nm (Figure 1a), while for Fe_3_O_4_@Au, the maximum absorption was highlighted at 538 nm (Figure 1b). Our results for the Fe_3_O_4_@Ag sample are concordant with those of Wibowo et al. (2021), who found the LSPR band maximum for a magnetite core–silver shell sample at 394 nm [36], while Guo et al. (2018) evidenced the maximum of similar composites at 420 nm [37]. Regarding the Fe_3_O_4_@Au sample, we found agreement with the results reported by Chen et al. (2016) for gold-coated magnetite nanoparticles, with a maximum absorbance at approximately 548 nm [38], and with those of Goon et al. (2009) who showed a maximum absorbance at 550 nm [39].

### 2.2. Results of Sample Investigation in Dark-Field Optical Microscopy

**Figure 2:**
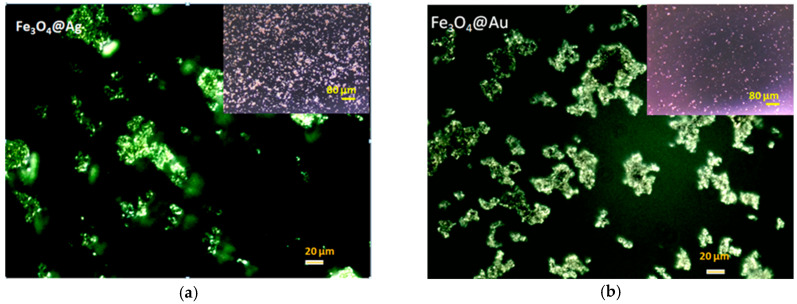
The results of optical investigation through dark-field imaging: (**a**) Fe_3_O_4_@Ag sample; (**b**) Fe_3_O_4_@Au sample (scale bar 20 µm, objective 40×). Inset image with 10× objective (scale bar 80 µm).

The investigation of sample drops dried on microscope slides has resulted in the images captured in the dark-field technique approach (Figure 2a,b). Due to LSPR phenomena, the presence of silver and gold could be clearly observed, suggesting the formation of plasmonic nanocomposites.

### 2.3. The Results of Electron Microscopy Investigation

Investigations by TEM highlighted the fine graining of the studied samples as well as their morphology, allowing for a size estimation.

We present the results of the TEM investigation for the two nanocomposite samples as well as for magnetite- and glucose-coated magnetite nanoparticles—before their treatment with silver and gold precursor reagents.

**Figure 3:**
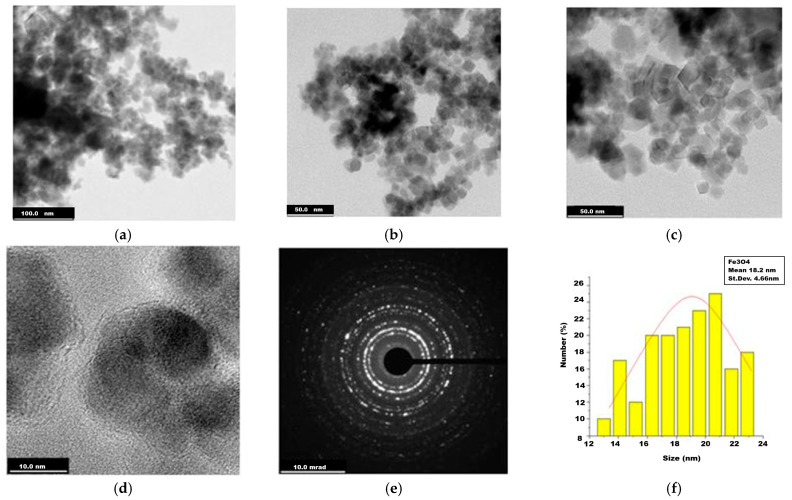
TEM images of the uncoated magnetite (Fe_3_O_4_): (**a**) 100 nm scale bar; (**b**,**c**) 50 nm scale bar; (**d**) 10 nm scale bar; (**e**) Selected Area of Electron Diffraction (SAED); (**f**) diameter histogram fitted with normal distribution curve.

In Figure 3, TEM images for uncoated magnetite can be observed, with symmetric particle shapes, quasi-spherical and polyhedral shapes, such as cubic. Some particle overlaps appear, which may be caused by the rather poor water dispersibility of pure nanoparticles without organic coating.

**Figure 4:**
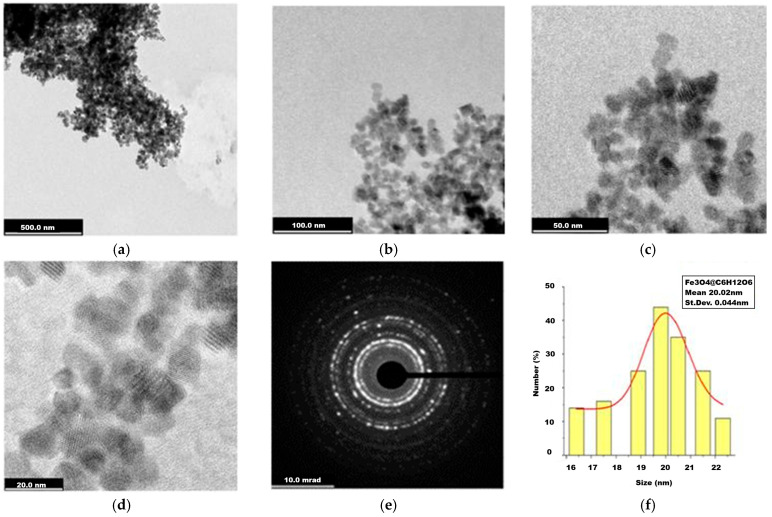
TEM images recorded for glucose-coated magnetite (Fe_3_O_4_@C_6_H_12_O_6_): (**a**) 500 nm scale bar; (**b**) 100 nm scale bar; (**c**) 50 nm scale bar; (**d**) 20 nm scale bar; (**e**) Selected Area of Electron Diffraction (SAED); (**f**) diameter histogram fitted with normal distribution curve.

The magnetite stabilized with glucose presents rather well-dispersed nanoparticles (Figure 4a–c) with nanometric sizes slightly larger than those of uncoated magnetite. Crystalline matter could be observed in Figure 4d–e, like in the case of uncoated magnetite (Figure 3d), which denotes crystal planes of electron diffraction, as confirmed by the XRD analysis below. The mean size of around 20 nm was estimated by the statistical approach of the particle size histogram (Figure 4f).

**Figure 5:**
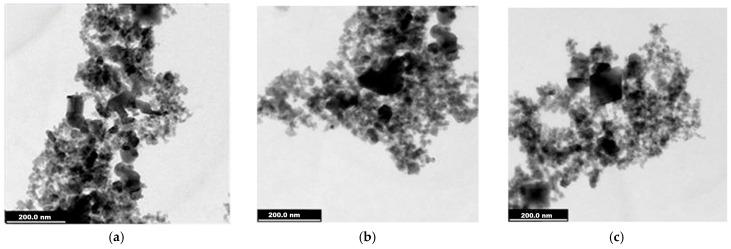

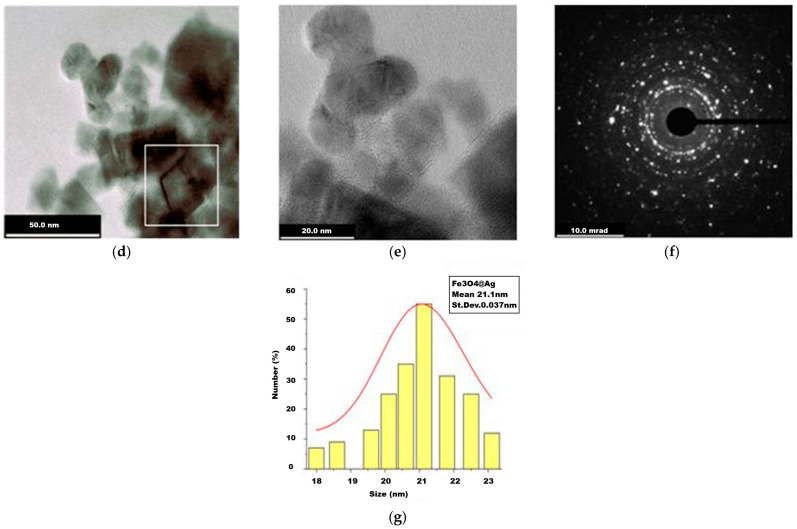
TEM images recorded for Fe_3_O_4_@Ag sample: (**a**–**c**) 200 nm scale bar; (**d**) 50 nm scale bar (white square marks a cubic structure); (**e**) 20 nm scale bar; (**f**) Selected Area of Electron Diffraction (SAED); (**g**) histogram fitted with normal distribution curve.

In Figure 5, the morphology of Fe_3_O_4_@Ag nanocomposites is given, with symmetrically formed individual nanoparticles (such as cubic ones, Figure 5d) having diameters of 18–23 nm and particle clusters up to 200 nm in size. The clustering of Fe_3_O_4_@Ag magnetoplasmonic particles is concordant with the images obtained in dark-field microscopy (Figure 2a), where particle clusters of hundreds of nm size are visible.

**Figure 6:**
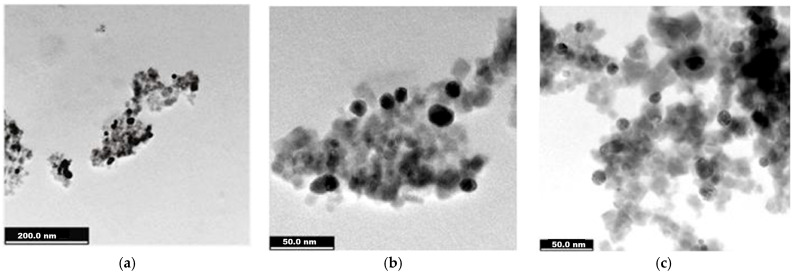

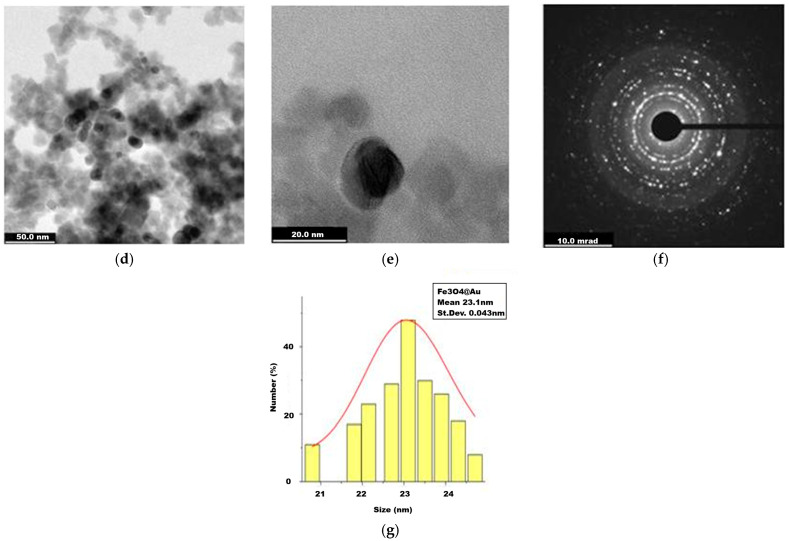
TEM imaging for Fe_3_O_4_@Au sample: (**a**–**c**) 200 nm scale bar; (**d**) 50 nm scale bar; (**e**) 20 nm scale bar; (**f**) Selected Area of Electron Diffraction (SAED); (**g**) histogram fitted with normal distribution curve.

The Fe_3_O_4_@Au sample gave TEM images (Figure 6) that presented better dispersed nanocomposites (as suggested by dark-field microscopy, Figure 2b) which were 21–25 nm in size and were mainly quasi-spherical (Figure 6a–e).

One might consider that some structures shown in gray tones could also be nanocomposites with noble metals located in a basal plane of the 3D deposition of the analyzed material in contrast to the nanocomposites located in the closest plane.

Measuring the particle size, we found the mean value of the nanocomposite physical diameters to be about 21 nm for Fe_3_O_4_@Ag and 23 nm for Fe_3_O_4_@Au (Table 1). The overlapping of some particles may represent the tendency to agglomerate in clusters in native suspensions or may be the result of deposition and drying on the TEM sample support. The SAED patterns shown in Figure 5f and Figure 6f confirm the crystalline nature of both nanocomposites, as evidenced by the presence of characteristic diffraction rings.

The contribution of silver and gold to the formation of magnetic nanocomposites was confirmed better by the results of EDS analysis.

### 2.4. The Results of EDS Investigation

**Figure 7:**
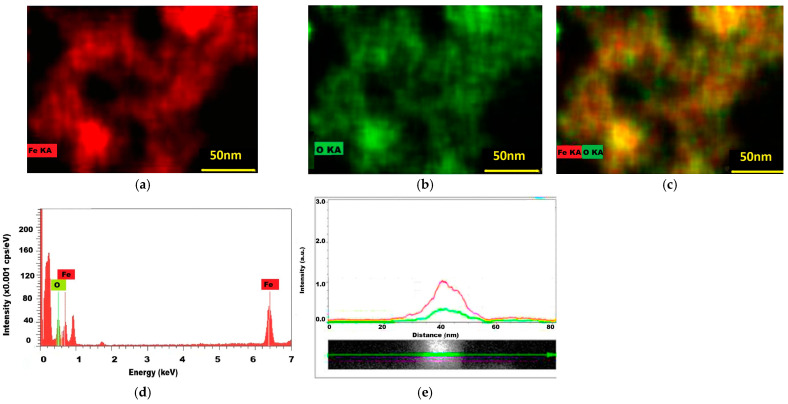
EDS analysis results of uncoated magnetite nanostructures: (**a**) iron distribution (red); (**b**) oxygen (green) distribution; (**c**) iron (red) and oxygen (green) distribution maps; (**d**) the EDS spectrum of magnetite sample (cps—counts per sec); (**e**) the distributions of iron and oxygen (red line—iron, green line—oxygen).

**Figure 8:**
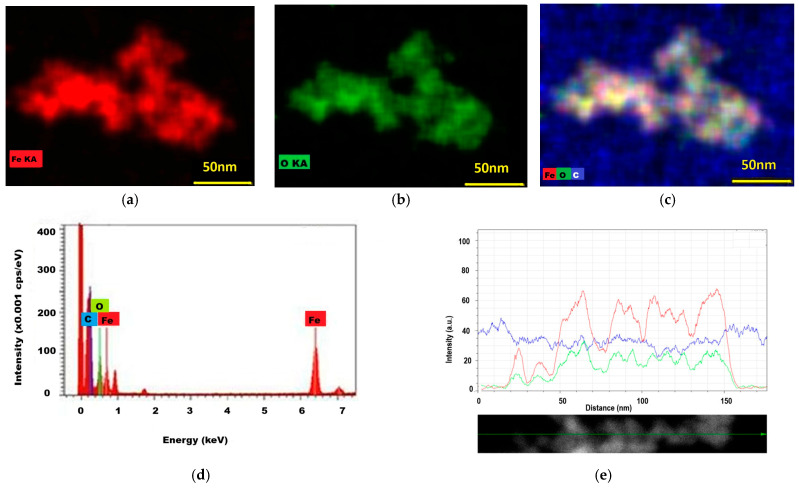
EDS analysis results of Fe_3_O_4_@C_6_H_12_O_6_ magnetic nanostructures: (**a**) iron distribution (red); (**b**) oxygen (green) distribution; (**c**) iron (red), oxygen (green), and carbon (yellow) distribution maps; (**d**) the EDS spectrum of magnetite sample (cps—counts per sec); (**e**) the distributions of iron, oxygen, and carbon from glucose in several aligned magnetic nanoparticles (red line—iron, green line—oxygen, and blue line—carbon).

The application of the EDS technique allowed for the identification of silver and gold through their atomic emission lines. First the uncoated magnetite is presented (Figure 7) with well-matched distribution maps for iron and oxygen. Next, the glucose-stabilized magnetite nanostructures were shown to present coherent distributions of iron and oxygen, as well as of carbon from the glucose-stabilizing molecules (Figure 8). There seems to be a perfect match of elemental distribution in the same area, as denoted by color spots—red (iron Kα emission line), green (oxygen Kα emission line) and blue (carbon Kα emission line).

The EDS investigation carried out on the uncoated magnetite produced iron and oxygen distribution maps well-matched on the same sample spot (Figure 7a–c), as generated by the two elements’ emission Kα lines. In the EDS spectrum, the iron and oxygen lines are present at 0.7 eV and 6.4 eV, respectively, and at 0.5 eV, accompanied by the carbon line (corresponding to air carbon dioxide or to the sample support).

The EDS spectrum of glucose-coated magnetite (Figure 8d) presents the emission lines of iron—the Kα line at about 6.4 eV and Lα line at about 0.7 eV—as well as the emission lines of oxygen (Kα at 0.5 eV) and carbon, mainly from glucose that was added during synthesis to stabilize the magnetite nanoparticles in suspension (Kα at about 0.2 eV). In Figure 8e, the intensities of the iron, oxygen, and carbon emission lines along several neighbor-aligned nanoparticles are presented.

**Figure 9:**
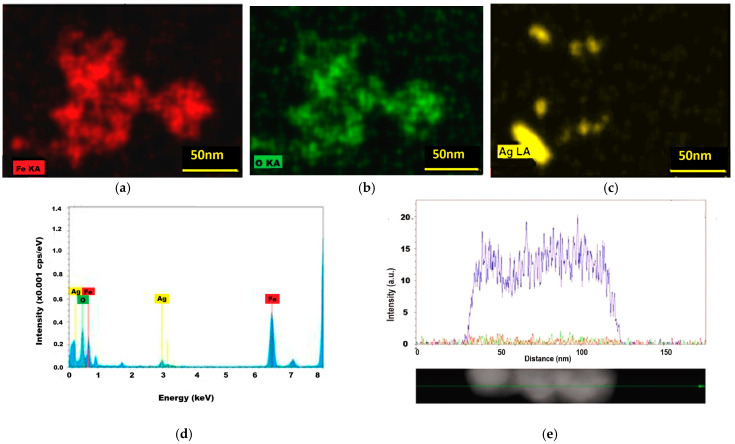
EDS analysis results of Fe_3_O_4_@Ag nanocomposites: (**a**) the map of iron distribution—red spots correspond to iron Kα line; (**b**) the distribution of oxygen ions—green spots correspond to oxygen Kα emission line; (**c**) silver distribution—yellow spots indicate silver through the Ag Lα atomic emission line; (**d**) the EDS spectrum for Fe_3_O_4_@Ag sample (cps—counts per second); (**e**) silver distribution (blue recording) on an aggregate of three nanocomposites (the presence of iron and oxygen is represented by red and green recording lines).

The characteristic emissions of silver (Lα line) were detected in the element map distribution (Figure 9c) and in the EDS spectrum of the Fe_3_O_4_@Ag nanocomposites (Figure 9d) at approximately 3 eV, accompanied by another silver signal at approximately 0.5 eV, close to that of carbon, in agreement with Krishnaraj et al. (2010) [40]. In Figure 9e, the silver distribution over a three-nanocomposite aggregate of Fe_3_O_4_@Ag is represented by the blue recording line. EDS analysis highlighted that the process of silver reduction with glucose at the magnetite nanoparticle surface seems to favor cluster formation from Fe_3_O_4_@Ag nanocomposites. This was also highlighted by the results of dark-field microscopy visualization (micrometer-sized clusters) and DLS results (some maxima in the micrometer range), while the study of magnetic properties demonstrated the contribution of non-magnetic materials to the decrease in saturation magnetization and magnetic core size in silver—and also gold—nanocomposites.

**Figure 10:**
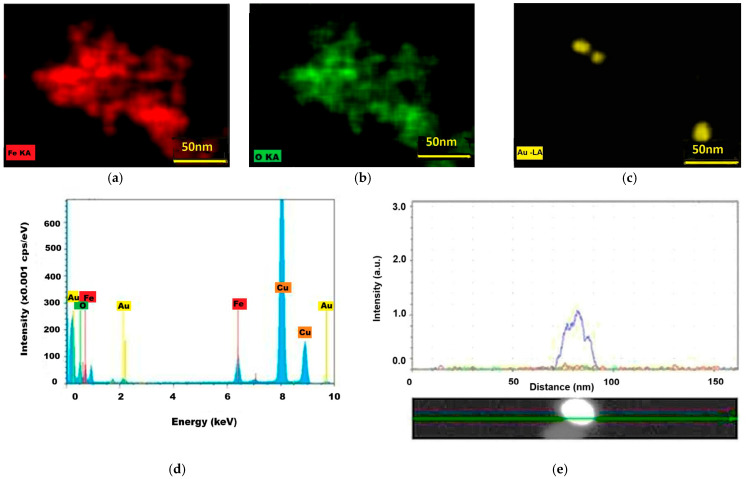
The results of EDS analysis of Fe_3_O_4_@Au sample: (**a**) the iron spatial distribution map (red spots corresponds to iron Kα emission line); (**b**) oxygen distribution (green spots corresponds to Kα line of oxygen atomic emission); (**c**) gold presence is marked by yellow color corresponding to Au Lα emission line; (**d**) the EDS spectrum for Fe_3_O_4_@Au sample (cps—counts per second); (**e**) gold distribution (blue recording line) on a nanoparticle of about 20 nm (iron—in red and oxygen in green).

For the Fe_3_O_4_@Au sample, the yellow spots corresponding to gold (Lα emission line), found in the same zones in the iron and oxygen maps (Figure 10a–c), are believed to indicate the gold attached to the magnetoplasmonic nanostructures.

The distribution of gold (Figure 10e) on a nanoparticle was given by the blue recording line, while iron and oxygen at the selected spot are given by red and green recording lines. The relative intensity of the gold maximum was considerable higher than that of iron, in accordance with Tonthat et al. (2023) [41]. The gold characteristic emission lines (Figure 10d) Lα1 at ~9.7 keV and Mα and Mβ lines at ~2 keV were recorded along with the iron Kα line at about 6.4 eV and the Lα line at about 0.7 eV, while the Cu strong lines at 8 and ~9 keV are given by the sample support.

### 2.5. XRD Analysis Results

**Figure 11:**
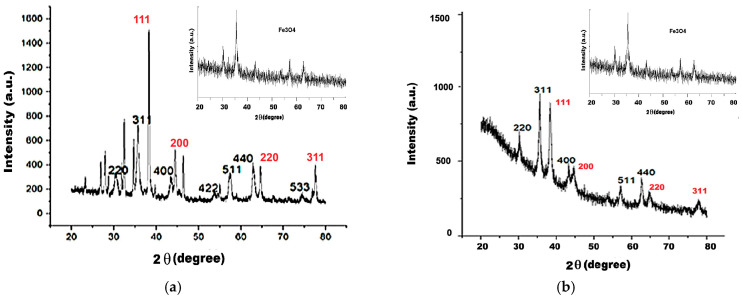
The results of XRD investigation; (**a**) for Fe_3_O_4_@Ag nanocomposites; in black—the magnetite diffraction peaks and in red—the silver diffraction peaks; (**b**) for Fe_3_O_4_@Au nanocomposites; in black—the magnetite diffraction peaks and in red—the gold diffraction peaks. The inset shows the Fe_3_O_4_@C_6_H_12_O_6_ XRD recording.

Crystalline structure of the studied samples was predicted in the SAED images from the TEM recordings (Figure 4e, Figure 5f and Figure 6f) but is better described by the XRD diagrams presented in Figure 11a,b.

In Figure 11, we present the diffractograms recorded for the studied nanostructures to show their crystalline properties. In Table 2, the characteristic peaks for our samples corresponding to magnetite, silver, and gold are provided.

These values were in accord with the standards from the literature for magnetite, silver, and gold (JCPDS No. 19-0629, JCPDS file No. 04-0783, and JCPDS file: 04-0784).

### 2.6. The Results of VSM Investigation

**Figure 12:**
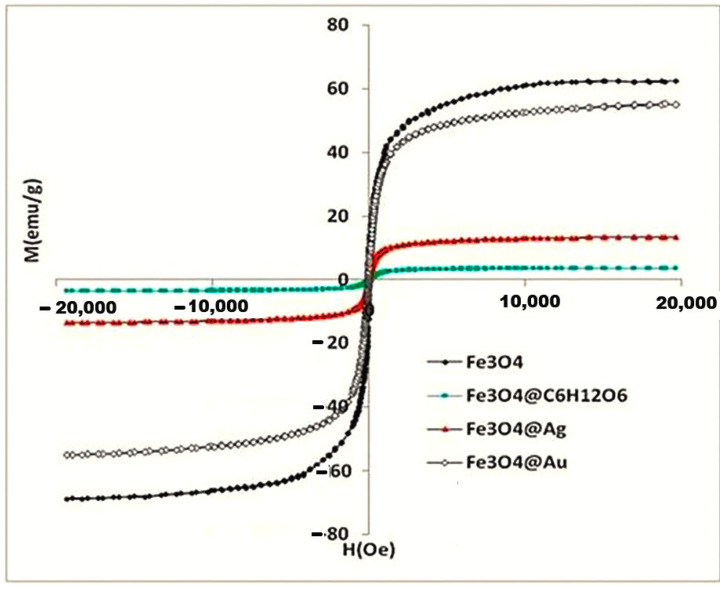
The hysteresis curves for the studied magnetoplasmonic nanocomposites compared to magnetite and glucose-coated magnetite.

The application of the VSM (Vibration Sample Magnetometry) technique resulted in the magnetization curves *M(H)*, for the two magnetoplasmonic samples in comparison to the magnetite ones, recorded at room temperature (Figure 12).

In Table 3, we also present the magnetic core diameters estimated according to Langevin’s theory with Formula (1) [42]:

(1) $$d_{M}={\left[\frac{18k_{B}T}{\pi \mu_{0}M_{s}\cdot M_{b}}{\left(\frac{dM}{dH}\right)}_{H\to 0}\right]}^{1/3}$$

where k_B_ is Boltzmann’s constant, µ_0_ is the magnetic permeability of the vacuum, and M_S_ and M_b_ are the saturation magnetizations of the sample and of bulk magnetite, respectively, while (dM/dH) is the slope of the M(H) curve at the origin of the graph. The saturation magnetization of bulk magnetite—according to the literature [42]—was the same for the magnetic core calculations of all the studied samples.

According to Table 3, the magnetic diameter d_M_ decreases significantly from 13.1 nm for the uncoated magnetite to 12 nm for the glucose-coated magnetite nanoparticles, to 9.6 nm for Fe_3_O_4_@Ag nanocomposites and, to 7.3 nm for Fe_3_O_4_@Au nanocomposites, evidencing that the non-magnetic phase is larger in the case of Fe_3_O_4_@Au compared to Fe_3_O_4_@Ag nanocomposites. Still, it appears that our magnetoplasmonic products could be useful in the implementation of theranostic procedure tools that involve responsivity to external magnetic gradients.

### 2.7. Results of Analysis by DLS (Dynamic Light Scattering) Method

**Figure 13:**
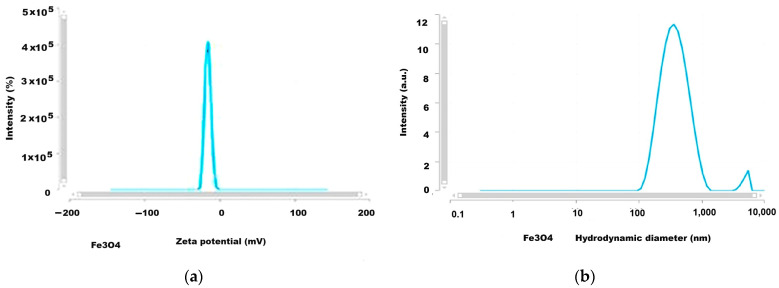
The results of DLS investigation for uncoated Fe_3_O_4_ nanoparticles: (**a**) Zeta potential; (**b**) hydrodynamic diameter (lin-log representation).

**Figure 14:**
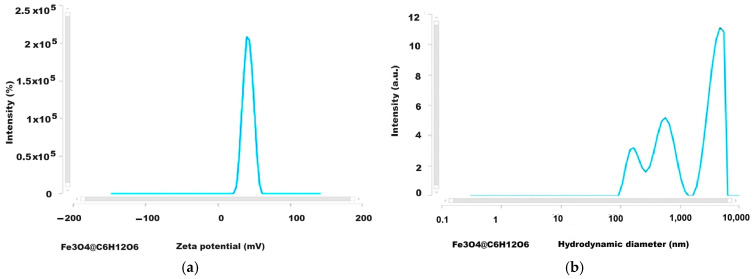
The results of DLS investigation for Fe_3_O_4_@C_6_H_12_O_6_ nanoparticles: (**a**) Zeta potential; (**b**) hydrodynamic diameter (lin-log representation).

**Figure 15:**
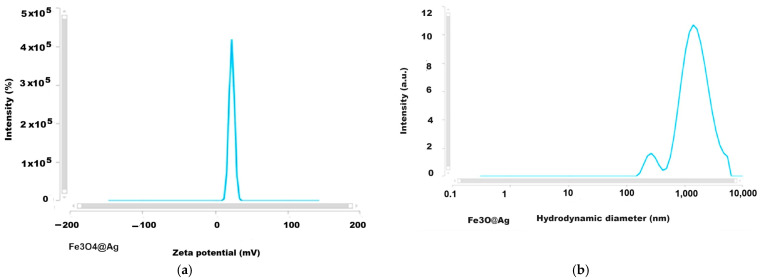
The results of DLS investigation for Fe_3_O_4_@Ag nanocomposites; (**a**) Zeta potential; (**b**) hydrodynamic diameter (lin-log representation).

**Figure 16:**
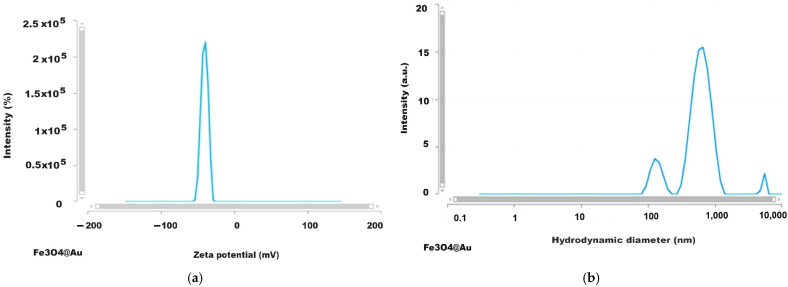
The results of DLS investigation for Fe_3_O_4_@Au nanocomposites: (**a**) Zeta potential; (**b**) Hydrodynamic diameter (lin-log representation).

The development of biomedical applications requires certain data on the behavior of nanoparticles in fluid media, such as biological environments, either intracellular or extracellular, which essentially involves the investigation of particles in dynamic motion in their water suspension by applying, for example, DLS investigation methods. The results we obtained for the Zeta potential and the hydrodynamic diameter are given in Figure 13, Figure 14, Figure 15 and Figure 16 and Table 4.

The Zeta potential values were −15.7 and +41.84 mV for magnetite nanoparticles uncoated or coated with glucose and were +23.05 mV and −40.57 mV for the magnetoplasmonic nanocomposites with silver and gold, respectively, denoting quite stable suspensions. From the viewpoint of the hydrodynamic diameter, the analyzed suspensions present multimodal distribution curves with the smallest mean diameter for the uncoated magnetite, suggesting sub-populations of nanoparticles.

### 2.8. Results of Raman Spectroscopy Investigation

**Figure 17:**
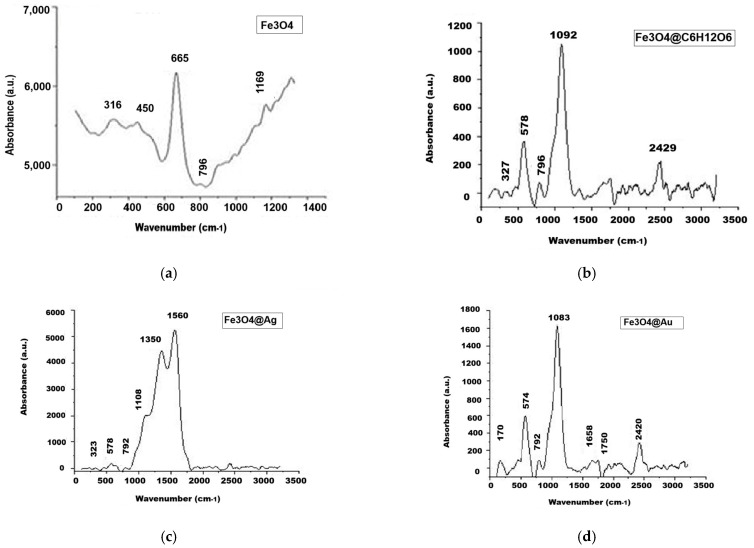
The Raman spectra for the studied samples: (**a**) Fe_3_O_4_; (**b**) Fe_3_O_4_@C_6_H_12_O_6_; (**c**) Fe_3_O_4_@Ag; and (**d**) Fe_3_O_4_@Au.

The results of the Raman spectroscopy analyses are presented in Figure 17. In all of the Raman spectra at wavenumbers under 800 cm^−1^, one can see the bands corresponding to Fe-O-bound vibrations, i.e., at 796 cm^−1^, 665 cm^−1^, 450 cm^−1^, and 316 cm^−1^ for magnetite nanoparticles (Figure 17a); at 796 cm^−1^, 578 cm^−1^, and 327 cm^−1^ for glucose-coated magnetite (Figure 17b); at 792 cm^−1^, 578 cm^−1^, and 323 cm^−1^ for Fe_3_O_4_@Ag nanocomposites (Figure 17c); and at 792 cm^−1^, 574 cm^−1^, and 170 cm^−1^ for Fe_3_O_4_@Au nanocomposites (Figure 17d). The presence of glucose molecules is revealed by the C-O vibration band at approximately 1090 cm^−1^.

These band assignments are consistent with Chavez et al. (2024) [43], Mohaček et al. (2022) [44], and Zhao et al. (2011) [45].

### 2.9. The Results of FTIR Analyses

**Figure 18:**
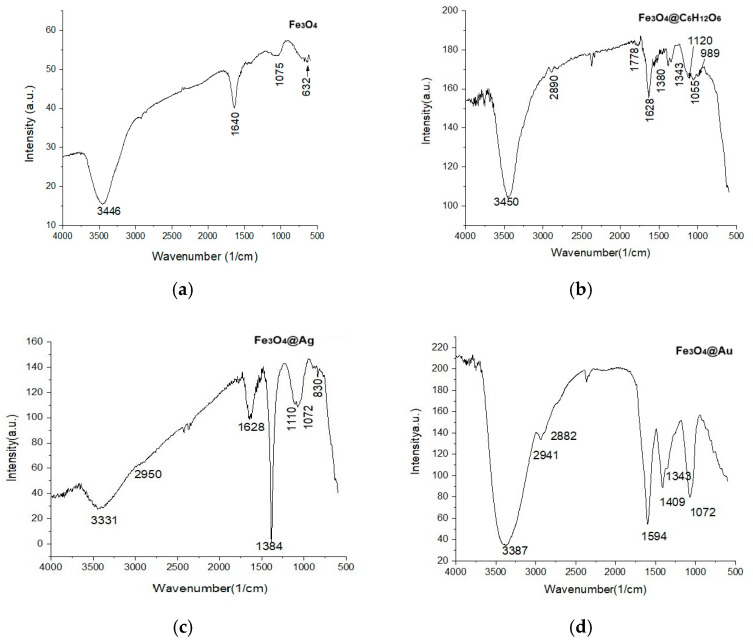
FTIR recordings for the studied samples: (**a**) Fe_3_O_4_; (**b**) Fe_3_O_4_@C_6_H_12_O_6_; (**c**) Fe_3_O_4_@Ag; and (**d**) Fe_3_O_4_@Au.

Figure 18 shows the FTIR spectra of Fe_3_O_4_ and Fe_3_O_4_@C_6_H_12_O_6_ nanoparticles, as well as of Fe_3_O_4_@Au and Fe_3_O_4_@Ag nanocomposites. The Fe-O vibrations could be identified with the maxima at 632 cm^−1^ (Figure 18), in accord with Chaki et al. (2015) [46], who found vibrations of the Fe-O bond in magnetite at the wavenumbers of 696–468 cm^−1^ or lower.

The main vibration frequencies and assignments are shown in Table 5.

The interactions between iron and oxygen, as well as those of carbon and oxygen, show bands that confirm the presence of magnetite and, respectively, of glucose in all of the studied samples.

### 2.10. Antimicrobial Activity Study. The Results of the Diffusimetric Method Application

**Figure 19:**
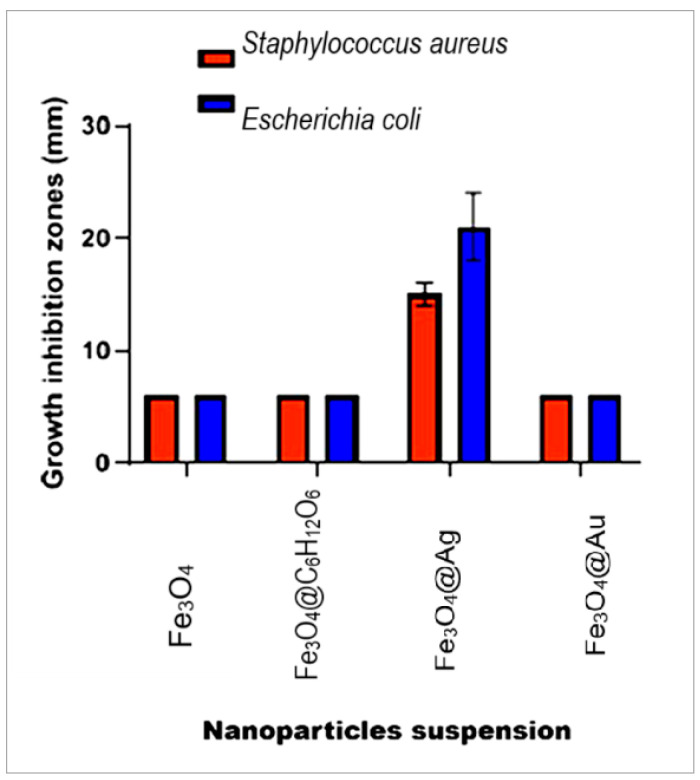
Diameters of the inhibition zones for *Escherichia coli* and *Staphylococcus aureus* treated with the nanoparticle suspension; the results represent the average of triplicates.

The results of the agar diffusion method are consistent with the dimensions of microbial growth inhibition zones after the administration of aliquots of nanoparticle suspensions (Figure 19). We obtained a relevant antimicrobial effect for the Fe_3_O_4_@Ag nanocomposites, differentiated according to the reference strains tested, with inhibition zones of significant size, as can be seen in Figure 19.

The diameters of the inhibition zones for the *Escherichia coli* strain were larger, with an average of 21 mm, compared to *Staphylococcus aureus*, where the average was 15 mm, indicating increased sensitivity of Gram-negative bacteria to Fe_3_O_4_@Ag compared to Gram-positive bacteria.

### 2.11. Antimicrobial Activity Study. Results of the Minimum Inhibitory Concentration Assessment

The minimum inhibitory concentration (MIC)—the lowest concentration of antimicrobial agent required to completely inhibit bacterial growth—was determined by reading the microtitration plates for each bacterial species tested (Table 6).

According to Table 6, the samples of Fe_3_O_4_@C_6_H_12_O_6_, Fe_3_O_4_@Au, and uncoated magnetite nanoparticles—which were also tested—induced inhibitory effects against the *Staphylococcus aureus* species at MIC values corresponding to the dilution factor of 8 or 16, with average MICs of 0.5 mg/mL, 1.479 mg/mL, and 2.430 mg/mL, respectively.

**Figure 20:**
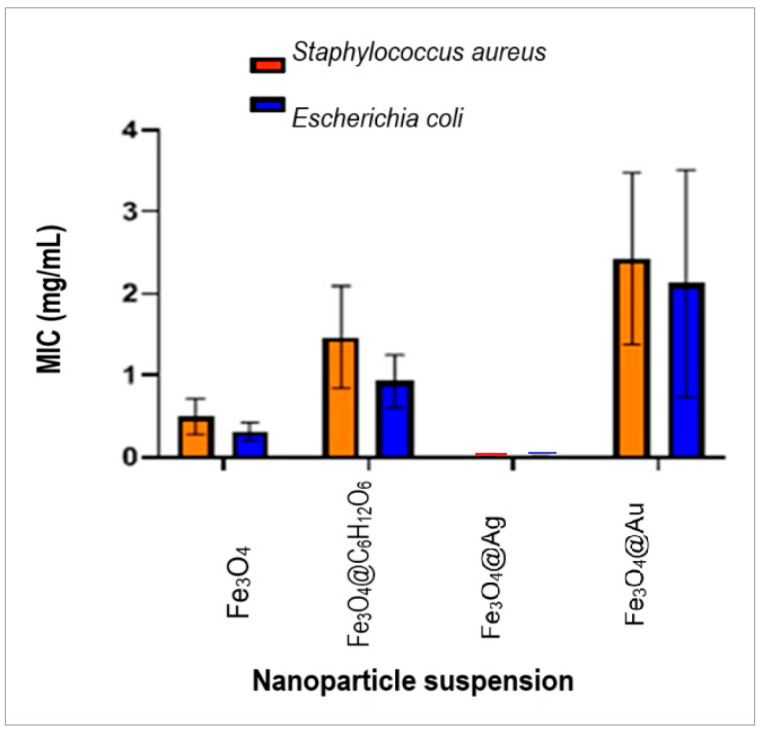
Graphical representation of the average MIC values (mg/mL) of the triplicates obtained for *Escherichia coli* and *Staphylococcus aureus* species in the presence of magnetic nanoparticles and nanocomposites. Error bars represent standard deviation.

In Figure 20, the remarkably strong inhibitory effect of Fe_3_O_4_@Ag nanocomposites on *Staphylococcus aureus* was illustrated with an average MIC of only 0.017 mg/mL at low suspension concentrations, corresponding mainly to the high dilution factors of 512 and 1024.

In *Escherichia coli*, the dilution factor corresponding to the MIC was 16–32 for Fe_3_O_4_ and Fe_3_O_4_@C_6_H_12_O_6_ nanoparticles, and 8–32 for Fe_3_O_4_@Au nanocomposites. The average MIC values were 0.312 mg/mL, 0.924 mg/mL, and 2.126 mg/mL, respectively (Figure 20).

Also, against these bacteria, as for *Staphylococcus aureus*, the Fe_3_O_4_@Ag nanocomposites revealed a stronger inhibitory effect at high dilution factors of 512, 1024, and 2048, corresponding to a low average MIC of 0.012 mg/mL.

Statistical analysis demonstrated that there are significant differences between the average MIC values, according to the values of *p* = 0.006 and F = 8.808 for the *Staphylococcus aureus* species and *p* = 0.028 and F = 5.172 for *Escherichia coli*.

The minimum bactericidal concentration of the tested nanocomposites represents the minimum essential quantity for the eradication of bacterial populations (Table 7).

**Figure 21:**
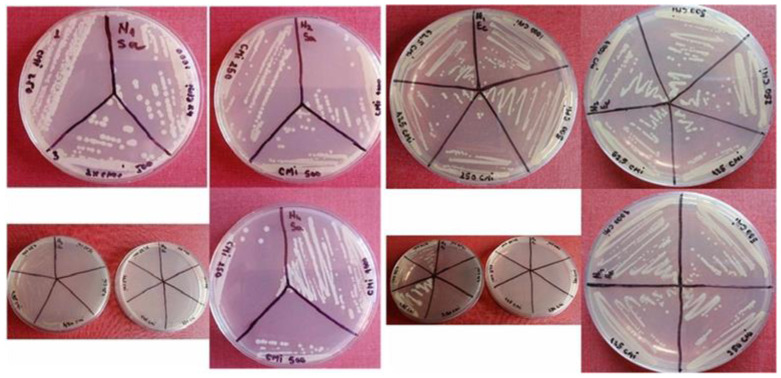
(**a**) Determination of the minimum bactericidal concentration (MBC) of magnetic nanocomposite suspensions against *Staphylococcus aureus*; (**b**) determination of the minimum bactericidal concentration (MBC) of magnetic nanocomposite suspensions against *Escherichia coli*.

The interpretation of the results for obtaining the MBC was achieved by reading the Petri plates (Figure 21a,b). The results corresponding to the magnetic nanoparticle suspensions against the *Staphylococcus aureus* species indicated that Fe_3_O_4_ nanoparticles, Fe_3_O_4_@C_6_H_12_O_6_ nanoparticles, and Fe_3_O_4_@Au nanocomposites do not show bactericidal activity for the concentrations used, but only inhibitory activity.

The MBC values were determined by inoculating the samples taken from the MIC test dilutions in which no bacterial growth was observed after incubation. In this way, the multiple sections of the Petri dishes reflect independent replicates of these inoculations and were used to confirm the bactericidal or bacteriostatic nature of the different concentrations of the compounds of interest tested. Regarding the bactericidal activity of the Fe_3_O_4_@Ag nanocomposites, it presented a strong bactericidal effect marked by a low average MBC value of 0.055 mg/mL, but also by the result of the ratio between the MBC and MIC which was less than 4, namely 3.20.

The results obtained by determining the bactericidal activity of magnetite nanoparticle suspensions against *Escherichia coli* revealed that Fe_3_O_4_ nanoparticles, Fe_3_O_4_@C_6_H_12_O_6_ nanoparticles, and Fe_3_O_4_@Au nanocomposites do not exhibit bactericidal properties for the tested concentrations, only inhibitory action.

## 3. Discussions

The application of various investigation techniques allowed us to highlight the plasmonic, microstructural, and magnetic properties of the studied samples. The absorption bands of the studied nanocomposites that originate from the phenomenon of localized surface plasmon resonance (LSPR) represent the response of nanostructured noble metals to incident light due to the oscillation motion induced in the metal conduction electrons; ferrophase alone only has a downward curve at relatively low wavelengths, with no specific maximum in the UV–Vis spectral recordings (Figure 1a,b). In the case of Fe_3_O_4_@Au nanocomposites, after the first treatment of ferrophase with chloroauric acid and glucose solution, the LSPR band was only slightly shaped but after two more treatments, the evidence of gold presence in the colloidal suspension was obtained (Figure 1a) as an absorption band with a maximum at 538 nm overlapped on the descendent tail given by ferrophase nanoparticles [47]. No further increase in absorption band was observed in the spectrum after subsequent administration of chloroauric acid. Thus, all investigations and analyses carried out on the Fe_3_O_4_@Au nanocomposites resulted from three treatments with chloroauric acid and also the tests for antimicrobial properties. The results we obtained in the study of the LSPR band of Fe_3_O_4_@Au nanostructures are consistent with those published in the literature, for example, by Xu et al. (2007) [48], who identified the spectral maximum at 534 nm, as well as by Iancu et al. (2020) [49], who found the LSPR band maximum at 542 nm.

The first treatment of the ferrophase with silver precursor salt and glucose solution revealed the characteristic absorption band at 398 nm (Figure 1b), while the subsequent addition of silver nitrate solution did not result in any significant increase in the UV–Vis spectrum. Therefore, all analyses for sample characterization and antimicrobial property testing were performed on the Fe_3_O_4_@Ag nanocomposites resulting from a single treatment with silver precursor solution.

In Figure 2, we provided the results of nanocomposite imaging in dark-field mode. This technique allows for the collection of the light scattered by the analyzed metallic object, due to the LSPR (localized surface plasmon resonance) phenomenon, favored by the properties of conduction electrons in noble metals capable of exerting oscillations in resonance with variations in the electric field of the incident light wave [50,51].

Consequently, in the microscope set in dark-field mode, the diffracted light can be selectively converted into distinct images that can be recorded and analyzed with a video camera and dedicated software.

The groups of bright spots shown in Figure 2a,b can be interpreted as plasmonic images of clusters of silver or gold magnetic nanocomposites—with micrometric sizes visualized in optical microscopy, while the underlying metal structures could be accurately measured in electron microscopy investigations exhibiting nanometric dimensions.

The overall image was also presented in the inset (10× objective, 80 µm scale bar) to show the higher tendency of agglomeration of the Fe_3_O_4_@Ag nanocomposites compared to the Fe_3_O_4_@Au nanostructures.

In the TEM records of the two magnetite nanoparticle samples, uncoated and glucose-coated ones were revealed to be composed of mainly individual particles with mean sizes around 18 and 20 nm (Figure 3 and Figure 4), while for the magnetite-derived nanocomposites (Figure 5 and Figure 6), some clusters of hundreds of nm could be observed. Along with the numerous grayscale structures, the images in the black scale could be highlighted, the former most likely indicating the simple ferrophase, while the latter shows the presence of noble metals attached to the magnetite nanoparticles. As for the nanocomposite morphology, rather symmetrical geometric shapes, quasi-spherical, or cubic ones were visualized for magnetite- and glucose-coated magnetite (Figure 3 and Figure 4) and also for the nanocomposites (Figure 5 and Figure 6), with a cubic structure being clearly evidenced in Figure 5d. TEM investigation revealed that Fe_3_O_4_@Ag nanostructures are rather agglomerated (Figure 5a–d), like evidenced previously by the dark-field microscopy (Figure 2a), while better dispersed particles or small clusters correspond to the Fe_3_O_4_@Au sample (Figure 6a–d). Statistical analysis of nanostructure dimensions resulted in the mean diameter of about 21 nm and 23 nm for Fe_3_O_4_@Ag and Fe_3_O_4_@Au, respectively. SAED images (Figure 3d, Figure 4e, Figure 5f, and Figure 6f) show electron diffraction planes suggesting the crystalline structure of the studied nanostructures.

In the diffractogram in Figure 11a, both magnetite and silver diffraction maxima were found in the Fe_3_O_4_@Ag nanocomposites. The diffraction peaks of magnetite were identified, as shown in Table 2, to be quite well matched to the standard peaks of the inverse spinel-type structure of cubic magnetite, according to JCPDS No. 19-0629. Compared to the Fe_3_O_4_ diffraction peaks, those of silver (with red in Figure 10a) correspond to silver’s crystallization into a face-centered cubic (fcc) structure (according to JCPDS file No. 04-0783) and show relatively high intensities, which denote a significant degree of silver attached to the magnetite in the Fe_3_O_4_@Ag nanocomposites [48,52]. It can be said that the ions provided by the silver precursor salt were efficiently converted into metal atoms on the surface of the magnetite cores [53].

At lower angles of around 27 degrees (Figure 11a), diffraction maxima were observed, possibly due to silver oxidization with Ag_2_O_3_ formation [54,55], and also possibly due to the small intensity peak of glucose (at about 28 degree) [56].

In Figure 11b and Table 2, the diffraction maxima for magnetite and gold are presented and describe the crystalline composition of the Fe_3_O_4_@Au sample.

The magnetite diffraction peaks also appear to be rather close to those described in the JCPDS No. 19-0629 files, while the gold peaks could be assigned to face-centered cubic (fcc) gold crystals, according to JCPDS file: 04-0784. The intensities of the gold diffraction peaks (in red) are comparable to those of magnetite, suggesting that the gold-containing magnetite particles are of approximately the same concentration as the magnetite ones. An amorphous phase also was evidenced at angles lower than 50 degrees (corresponding to the sample glass support), there was also more pronounced background noise than in the case of silver nanocomposites.

We obtained higher diffraction peaks for gold than for magnetite in the Fe_3_O_4_@Au sample. It could be because gold was added repeatedly to the reaction mixture during synthesis while silver was added just once (to obtain distinguishable absorption maxima characteristics of the two noble metals). Xu et al. (2007) found that the intensity of magnetite peaks decreases with the increase in the intensity of the gold/silver peaks, and this behavior proves that gold/silver ions were indeed reduced to the magnetite surface [48], while Iglesias-Silva et al. (2007) reported that total coverage of magnetite with silver led to the disappearance of magnetite diffraction peaks [52].

The magnetic properties of magnetite and glucose-coated magnetite nanoparticles and of the two magnetoplasmonic nanocomposite samples were presented in Figure 12 and Table 3.

All four magnetization hysteresis loops are very narrow, with remanent magnetization (*Mr*) values of 4.3, 2.19, 0.63, and 0.42 emu/g for Fe_3_O_4_, Fe_3_O_4_@C_6_H_12_O_6_, Fe_3_O_4_@Ag, and Fe_3_O_4_@Au, respectively. Accordingly, the coercive fields (*Hc*) have small values of 21.12 and 30.42 Oe for Fe_3_O_4_ and Fe_3_O_4_@C_6_H_12_O_6_, and of 23.80 Oe for Fe_3_O_4_@Ag and 74.81 Oe for Fe_3_O_4_@Au (Table 3). The very small width of hysteresis indicates that the analyzed samples exhibit dominant superparamagnetic behavior at room temperature. This is based on the hypothesis of round, uniform, and small size particles that align uniformly and in parallel with the applied magnetic field. But particle clustering affects this by decreasing saturation magnetization because magnetic interactions are no longer ideal. The non-magnetic material at the nanoparticle surface has the same effect. Thus, the saturation magnetization and magnetic diameter decreased for silver and gold nanocomposites—clustering was already visualized in both cases, but more so for silver, while the amount of non-magnetic material was higher for gold (the Fe_3_O_4_@Au nanocomposites being synthesized with a triple gold precursor supply).

The saturation magnetization (*Ms*) of Fe_3_O_4_ nanoparticles of 61.8 emu/g and of 55 emu/g for Fe_3_O_4_@C_6_H_12_O_6_ is consistent with the literature for similar magnetic nanoparticles synthesized by co-precipitation. For instance, Darmawan et al. (2023), reported 54.2 emu/g for magnetite and 18.8 emu/g for silver-covered magnetite (at 15 kOe external magnetic field) [57]. We found that the presence of silver or gold reduces the specific saturation magnetization (per unit mass) to 13.46 emu/g and 3.55 emu/g, for Fe_3_O_4_@Ag and Fe_3_O_4_@Au, respectively. Tonthat et al. (2023) obtained, for the saturation magnetization, values of 52.4 emu/g in the case of magnetite (at 30 kOe external magnetic field) and 9.7 emu/g in the case of gold-coated magnetite nanoparticles [41].

This is because the non-magnetic metal attached at the magnetic cores leads to an increase in interparticle separation, thus reducing the magnetic dipole–dipole interaction. The glucose content could be higher in the case of magnetite–gold nanocomposites because three successive treatments with a glucose solution were performed during synthesis. All these results recommend our magnetoplasmonic nanocomposites as promising candidates for implementation in theranostic procedures involving responses to properly adjusted external magnetic gradients.

DLS analyses that were carried out of the uncoated magnetite dispersed in water (Figure 13a,b) revealed the Zeta potential of −15.7 mV and the mean hydrodynamic diameter of 343 nm. According to the DLS investigation (Figure 14 and Table 4) the Zeta potential for the magnetite nanoparticles coated with glucose was rather high compared to the theoretical threshold of ±30 mV, reaching over 41 mV (Table 4), which indicates very good stability of the colloidal sample, and, as expected, it was better than the value for uncoated magnetite with a Zeta potential of −15.7 mV. Also, the Fe_3_O_4_@Ag sample (Figure 15a) presents a positive Zeta potential, which was relatively lower than for the magnetite sample, but still revealed rather good stability, i.e., 23 mV [58].

The Fe_3_O_4_@Au nanostructures were characterized by a negative Zeta potential (Figure 16a) of approximately −40mV, which ensures very good suspension stability, according to other authors [59,60], who reported negative values for gold-coated magnetite nanoparticles in different media.

The plot of the hydrodynamic diameter values of the uncoated magnetite highlighted the main maximum at approximately 300 nm with 12 times higher intensity than the secondary peak at about 103 nm (Figure 13b), which is not unusual given the lack of organic ingredients involved in the particle’s stabilization, while for the glucose-coated magnetite nanoparticle (Figure 14b), the graph is a multimodal distribution curve with a relatively low maximum for values of hundreds of nm and two larger maxima for values above a thousand nm. These are concordant with the results reported by Keerthana et al. (2015) for magnetic nanoparticles synthesized by the hydrothermal method and coated with glucose, which presented a relatively low maximum for the hydrodynamic diameter values of hundreds of nm along with higher maximum for a couple of thousands of nm, and the Zeta potential of 28.60 mV [61]. Also, for the studied nanocomposites, the DLS investigation results revealed multimodal curves of the hydrodynamic diameter values (Figure 15b and Figure 16b) with *Dh* ranging from hundreds of nm to over a thousand nm. The hydrodynamic diameter is the effective diameter of a particle in a liquid medium, including the solvent layer surrounding it, and since in our samples, the agglomeration tendency was highlighted—by dark-field microscopy and TEM—this seems to explain the dimensional subpopulations, as revealed by the *Dh* measurements. However, the electrical charges at the surface of the nanoparticles confer Zeta potentials capable of ensuring the stability of the samples. Since in our synthesis procedures’ stabilization in water was ensured with glucose, we assume that the interaction of these molecules with the surface of nanoparticles and nanocomposites favors the formation of relatively large aggregates (Table 4) which define dimensional subpopulations within the studied samples. In the case of uncoated magnetite, there is also a small maximum (12 times smaller than the main peak, Figure 13b) for particles or particle aggregates that are not unusual regarding the lack of organic ingredients for the particle’s stabilization. The polydispersity index of the Fe_3_O_4_@Ag nanocomposite suspension was relatively low, 0.313 in average, evidencing a rather monodispersed sample (PDI ≤ 0.3 corresponds to monodisperse or mostly monodisperse particles) [62].

Other authors studying silver magnetite nanocomposites have also found a multimodal graph, with four maxima for the hydrodynamic diameter size distribution, positioned from a hundred to several thousand nm [63].

The value of the hydrodynamic diameter, *D_h_*, averaged for all sub-populations as resulting from three consecutive measurements (Table 4); for Fe_3_O_4_@C_6_H_12_O_6_ nanoparticles it is, on average, 974 nm compared to 343 nm for the uncoated magnetite, while for Fe_3_O_4_@Ag nanocomposites the values of *D_h_* increase, being, on average, 1193 nm, while for Fe_3_O_4_@Au nanocomposites the average value was 579 nm. The polydispersity index (PDI) resulted for Fe_3_O_4_, Fe_3_O_4_@C_6_H_12_O_6_, and Fe_3_O_4_@Au in values 0.274, 0.55, and 0.94 larger than for Fe_3_O_4_@Ag but still resulted stable suspensions due to electrostatic repulsion, described by the Zeta potential’s magnitude.

The Raman spectrum for the uncoated magnetite is presented in Figure 17a. According to Hai et al. (2008), it should contain the typical vibration modes at 670 cm^−1^, 535 cm^−1^, and 306 cm^−1^ [64], which can be identified in our graph with the vibrational peaks at 665 cm^−1^, 450 cm^−1^ (next to a shoulder around 500 cm^−1^), and 316 cm^−1^ (Figure 17a). In all of the three Raman spectra corresponding to glucose-coated magnetite and to nanocomposites containing silver and gold, we identified the Fe-O vibrations. In Figure 17b, for glucose-coated magnetite, they are at 796 cm^−1^, 578 cm^−1^, 327 cm^−1^ (shoulder), i.e., increasing the values of the wavenumbers compared to Figure 17a.

In Figure 17c, these peaks were highlighted at 792 cm^−1^, 578 cm^−1^, and 323 cm^−1^, corresponding to Fe_3_O_4_@Ag nanocomposites, while in Figure 17d they are at 792 cm^−1^ and 574 cm^−1^, for Fe_3_O_4_@Au nanocomposites, with a smallest peak at 170 cm^−1^. In the case of Fe_3_O_4_@Ag, the Fe-O vibration maxima are less intense, and in this range, nanosilver has weak vibrations too [65], which can interfere with those of magnetite, making it possible that the degrees of freedom of vibrational movement in magnetite are limited due to the binding of silver to magnetite. The organic component (glucose) involved in the preparation of nanoparticles is revealed by the vibration of the C-O bond, i.e., the maximum at approximately 1090 cm^−1^. In the case of the Fe_3_O_4_@Ag sample (Figure 17c), this vibration is represented by a shoulder, as the nearby Ag presents two intense and broad maxima at 1350 cm^−1^ and 1560 cm^−1^ [66]; the first tends to partially overlap with the glucose band at 1108 cm^−1^. Also, these vibrations appear in Fe_3_O_4_@Au (Figure 17d) at almost the same wavenumbers as for the Fe_3_O_4_@Ag sample, but with much lower intensities compared to the glucose vibrations, suggesting that gold, compared to silver, interacts with magnetite significantly less. Thus, the surface modifications of the magnetic cores were confirmed by means of the Raman spectra.

In the FTIR recordings from Figure 18, the Fe-O vibrations could be identified with the maxima at 632 cm^−1^, in accord with Chaki et al. (215) [46], who found the vibrations of the Fe-O bond in magnetite at the wavenumbers of 696–468 cm^−1^ or lower. In our case, it was only for uncoated magnetite that the Fe-O vibrations could be identified, while for the other samples, where glucose was involved, there vibrations that dominated the range of small wavenumbers overlapping on the metal–oxygen peaks. The peak at 1075 cm^−1^ evidenced in the FTIR spectrum of uncoated magnetite was mentioned by several authors as also representing the vibrations of metal–oxygen bonds [67,68], but in the presence of glucose it is hidden under the stronger vibrations of C-O and C-C bonds.

The C-O and C-C bonds are supposed to provide vibrational bands at 1055 cm^−1^ (for Fe_3_O_4_@C_6_H_12_O_6_) and 1070 cm^−1^ (for Fe_3_O_4_ and Fe_3_O_4_@Ag), like in Table 5, while the maxima at 1385 cm^−1^ (for Fe_3_O_4_ and Fe_3_O_4_@Ag) and 1409 cm^−1^ (for Fe_3_O_4_@Au) could be attributed to the deformation vibrations corresponding to the C-O-C and C-O-H bonds—due to the glucose to which the gold and silver bind at the surface of the nanoparticles [69].

According to Suvarna et al. [70], the adsorption of glucose on the surface of gold and silver is confirmed by the presence of well-defined bands in the glucose fingerprint range between 1000 and 1600 cm^−1^.

The deformation vibrations of the O-H bonds in water molecules (Figure 18, Table 5), which were adsorbed on the surface of the nanoparticles, were assigned to the vibration bands at 1628 cm^−1^, 1598 cm^−1^, and 1640 cm^−1^ [24,49].

Also, the low-intensity vibration at 2314 cm^−1^ may correspond to carbon dioxide (Figure 18, Table 5) from air dissolved in the reaction medium, which was subsequently adsorbed on the surface of the iron oxides [21,68].

Symmetric and antisymmetric stretching vibrations of hydroxyl, OH, corresponding to the traces of water were highlighted in the spectral range from 3300 to 3700 cm^−1^, according to Safi et al. (2015) [71], but to the same extent, the broad bands in this range could also include the stretching vibrations of the CH bonds in glucose.

The antibacterial activity of the nanoparticles and nanocomposites described above was investigated by specific microbiological methods. The bacteria growth inhibition (revealed by the transparent zones of the agarized culture medium around the paper disks impregnated with nanoparticle suspensions) was the main indicator of the tested microorganisms’ susceptibility to the tested samples.

Since the zones of growth inhibition for the glucose-stabilized Fe_3_O_4_ suspension (Fe_3_O_4_@C_6_H_12_O_6_), for the uncoated Fe_3_O_4_ nanoparticles, and for the Fe_3_O_4_@Au nanocomposites did not exceed the 6 mm diameters of the paper disks, their growth inhibition activity could not be estimated. This was found for both *S. aureus* and *E. coli,* which did not show clear translucent halos that exceeded the nanoparticle-impregnated paper disks. The clear and distinct inhibition zones given by Fe_3_O_4_@Ag nanocomposites (Figure 19) of 15 and 21 mm for *S. aureus* and *E. coli*, respectively, reflect the susceptibility of both of the tested germs. Also, Shatan et al. (2019) evidenced the susceptibility of *E. coli* to magnetite/silica/silver nanocomposites with granulation and magnetizability similar to those of our nanocomposites (21 nm diameter and 26 emu/g saturation magnetization) [72].

Along with the diffusion method that allowed for a semi-quantitative estimation of the antimicrobial potential of the tested nanoparticles, the quantitative assay was performed by the estimation of the MIC (minimum inhibitory concentration) and MBC (minimum bactericidal concentration).

All of the nanoparticles and nanocomposites tested appear to be able to inhibit the growth of *E. coli* bacteria, with mean minimum inhibitory concentration (MIC) values ranging from 2.30 mg/mL for Fe_3_O_4_@Au to 0.01 mg/mL for Fe_3_O_4_@Ag (and intermediate values of 0.31 mg/mL for Fe_3_O_4_ and 0.92 mg/mL for Fe_3_O_4_@C_6_H_12_O_6_). In the case of inhibiting the growth of *S. aureus*, the lowest mean minimum inhibitory concentration (MIC) value was 0.02 mg/mL for Fe_3_O_4_@Ag and the highest was 0.43 mg/mL for Fe_3_O_4_@Au nanocomposites (with 1.47 mg/mL for Fe_3_O_4_@C_6_H_12_O_6_ and 0.5 mg/mL for Fe_3_O_4_ uncoated nanoparticles). The best antimicrobial activity was demonstrated by the Fe_3_O_4_@Ag nanocomposites, with lowest MIC values in the case of both Gram-positive and Gram-negative microorganisms.

In our experimental study, the results showed that the minimum inhibitory concentration (MIC) values of Fe_3_O_4_@Au nanocomposites were higher than those of Fe_3_O_4_ nanoparticles, either coated or uncoated with glucose, for both types of bacteria.

Thus, it is intriguing how the presence of gold on the surface of magnetite nanoparticles can modify the antimicrobial activity of magnetite, and we believe that this could be related to the blocking action of gold for iron ions at the magnetite surface.

If the gold attached to the surface of iron oxide nanoparticles is in relatively small amounts and does not constitute a compact shell, then it can still allow the action of iron ions and also exert its own antimicrobial action—depending on the susceptibility of the bacteria it interacts with. If the surface of the magnetic nanoparticle is heavily coated with gold, then this may block the activity of iron ions, so that magnetic nanocomposites with gold on the surface may exhibit a reduced antimicrobial effect compared to the original magnetic nanoparticles. This could also be the case for our experimental results. In the comparative study conducted by Chatterjee and collaborators in 2011 [7], for magnetite nanoparticles and gold nanoparticles, it was demonstrated that the treatment of *E.coli* bacteria with different concentrations of gold nanoparticles (25 μg/mL, 50 μg/mL, 75 μg/mL, and 100 μg/mL) did not generate significant changes in the growth curve, highlighting the non-toxic nature of gold nanoparticles in the bacterial system.

The sensitivity of the Gram-negative bacterium *E. coli* to Fe_3_O_4_@Ag suspensions, given by the lowest mean values of the minimum inhibitory concentration (MIC), proved to be higher than that of the Gram-positive bacterium *S. aureus*, since for all suspensions, the latter required higher mean values of the MIC.

Thus, the results obtained emphasize the correlation between the structure of the cell walls of the microorganisms and the mechanism of action of the nanoparticles, which have a better interaction with the outer membrane of Gram-negative bacteria than with the cell wall of Gram-positive bacteria, which behaves as a protective barrier due to the thick peptidoglycan layer. A study published by Sharaf et al. (2022) [27] shows that Fe_3_O_4_4@Ag nanocomposites tested against *E. coli* by the diffusimetric method led to microbial growth inhibition zones of 15 mm in diameter, a value concordant with our results of a 20 mm inhibition zone. The value of the MIC indicator obtained by those authors was 5.4 μg/mL (0.0054 mg/mL), tenfold less in comparison with the order of magnitude obtained in our case.

Park et al. (2019) [25] synthesized magnetite nanoparticles stabilized with dextran and coated with silver that were tested for antimicrobial activity against *E. coli* by measuring the optical density of liquid suspensions inoculated with bacteria. Optical density, as a measure of the cell concentration in the liquid culture medium, determined the strongest effect for the concentration of 200 μg/mL of nanoparticles, which almost completely destroyed the microorganisms within 24 h of incubation at 37 °C.

Demonstrating the antimicrobial activity of a substance involves estimating the minimum inhibitory concentration value, as well as establishing the minimum bactericidal concentration (MBC), which represents the minimum concentration of a substance capable of destroying ≥99.9% of the bacterial inoculum. According to the literature, when the ratio between the MBC and MIC is less than or equal to four, this corresponds to a bactericidal effect, demonstrating that the eradication of the bacterial population occurs at a concentration close to that which determines growth inhibition. However, if this ratio is greater than four, it indicates a bacteriostatic effect, which means that concentrations significantly higher than those determining inhibitory activity are required to induce the death of microorganisms. This method is very important in the context of evaluating the antimicrobial capacity of magnetic nanoparticles, because it makes it possible to differentiate between the bacteriostatic and bactericidal activity manifested by them.

Regarding the bactericidal properties of the Fe_3_O_4_@Ag nanocomposites, they showed a strong effect, marked by a low average MBC value of 0.027 mg/mL, but also by the result of the ratio between the MBC and MIC, which was less than 4 (precisely, 2.28). The average MBC value was higher in the case of Gram-positive species compared to Gram-negative bacterial species, 0.055 mg/mL for *S. aureus* and 0.027 mg/mL for *E. coli*, respectively, which highlights a greater sensitivity of Gram-negative bacteria to low concentrations of Fe_3_O_4_@Ag nanocomposites compared to Gram-positive bacteria.

The magnetite/carbon/silver nanocomposites synthesized by Xia et al. (2011), in the form of magnetite spheres of over 100 nm decorated with AgNPs of approximately 15 nm through carbon, were characterized by a saturation magnetization of approximately 30 emu/g, and their antimicrobial activity was tested on the microorganism *E. coli*, by counting the surviving microbial colonies in agarized (solid) culture medium, finding a bactericidal effect of approximately 95% for a concentration of 5 μg/mL [73].

Studies by Park et al. (2019) showed that silver nanoparticles interact directly with the bacterial membrane through thiol groups (-SH) in their protein components, so they can disrupt the integrity of the membrane to the point of partially damaging it (leading to irreversible leakage of the cytoplasm) [25].

Silver ions can also be released (originating from silver atoms on the surface of nanoparticles and re-oxidizing in contact with the biological environment), which penetrate the cell membrane, and their cytotoxic effects result from the production of oxygen free radicals (ROS) with lethal effects on microorganisms.

The interest in such research is motivated by the possibility of improving the targeted delivery of antiseptic agents by applying an external magnetic field to which the magnetic component is responsive.

## 4. Materials and Methods

### 4.1. Reagents

The reagents required to synthesize the nanoparticles were as follows: ferric chloride (FeCl_3_ × 6H_2_O), ferrous sulfate (FeSO_4_ × 7H_2_O), sodium hydroxide (NaOH), D-glucose (C_6_H_12_O_6_), silver nitrate (AgNO_3_), and chloroauric acid (HAuCl_4_), and were obtained from Sigma-Aldrich (Merck Romania SRL, an affiliate of Merck KGaA, Darmstadt, Germany).

For testing the antimicrobial activity of the nanoparticles, the ATCC collection reference strains we used were *Staphylococcus aureus* ATCC 25923 and *Escherichia coli* ATCC 25922.

### 4.2. Synthesis of Magnetite/Silver and Magnetite/Gold Nanocomposites

To produce magnetite nanoparticles, a co-precipitation method adapted from Massart [74] was applied, with some modifications. The solutions of ferric and ferrous precursor salts were prepared using FeCl_3_ × 6H_2_O and FeSO_4_ × 7H_2_O (Table 8) in their stoichiometry (2:1) and were heated under magnetic stirring on a hot plate at a temperature of 65 °C until complete dissolution.

Subsequently, the hot sodium hydroxide solution was slowly added and the mixture was left under mechanical stirring at 85 °C, thus resulting in the black magnetic mud. High reaction temperature and slow feeding of the alkaline agent favored the formation of relatively small nanoparticles and also avoided the formation of pores at their surface.

The synthesized nanoparticles were resuspended in 250 mL of water and the sample was referred to as Fe_3_O_4_. Using a permanent magnet to separate the magnetic phase, the nanoparticle suspension was washed three times with hot water (300 mL in total) to remove impurities or unreacted salts.

The Fe_3_O_4_@C_6_H_12_O_6_ nanoparticle sample was finalized by stabilization in deionized water with glucose, according to Iancu et al. (2020) [49], using equal volumes of glucose 0.1 M and Fe_3_O_4_ suspension, as determined after several tests, which were allowed to react for one hour at approximately 85 °C under mechanical stirring; glucose acted as a coating agent, but did not contribute significantly to the resulting nanoparticle diameter [49].

The suspension of a magnetoplasmonic nanocomposite based on magnetite and gold, referred to as the Fe_3_O_4_@Au sample, was prepared by gold reduction with glucose on the magnetite nanoparticle surface according to [49], with several modifications. A volume of 10 mL of the Fe_3_O_4_ suspension (which was first sonicated in an EMAG Ultrasound Emmi 40HC device, EMAG-AG, Germany) completed with 20 mL of water was mixed and supplied with 3 mL of 1% chloroauric acid (HAuCl_4_); the mixture was heated at about 85 °C for 30 min under mechanical stirring and then sonicated at 75 Hz for 15 min. In the next step, 30 mL of glucose solution and 3 mL of chloroauric acid were added under mechanical stirring for 30 min, then the solution was sonicated for 15 min. Glucose was added to ensure the controlled reduction of gold into Fe_3_O_4_ nanoparticles [52]. The process was repeated twice, thus obtaining the Fe_3_O_4_@Au hybrid nanostructures according to UV–Vis recorded monitoring.

The suspension of a magnetoplasmonic nanocomposite based on magnetite and silver was referred to as the Fe_3_O_4_@Ag sample and was prepared from 10 mL of a suspension of Fe_3_O_4_ nanoparticles, it was sonicated at 75 Hz for 15 min, to which 10 mL of glucose solution was added (adapted after [49]); then, the mixture was left for 15 min at 85 °C under mechanical stirring. Finally, 10 mL of silver nitrate solution was added and the mixture was kept under mechanical stirring for one hour.

### 4.3. Nanoparticles Characterization Methods

The spectral monitoring of nanoparticle formation was accomplished with a UV–Vis device, Shimadzu PharmaSpec 1700 (Shimadzu Corporation, Kyoto, Japan), using quartz cuvettes of 1 cm optical path. The absorption spectra were recorded and processed with UV-Probe software, version 2.71.

The capture and analysis of dark-field microscopic images was performed with an OPTIKA B-383DK microscope (Bergamo, Italy) with 40× and 10× objectives while the DLT CamLite (Delta Optical, Poland) visualization software was used to analyze the images.

The granularity investigation was carried out using an Ultra-High Resolution Scanning Transmission Electron Microscope (UHR-TEM LIBRA^®^200MC) (Carl Zeiss GmbH, Oberkochen, Germany) with an EDS (energy dispersive spectroscopy) capability attached, while particle size measurements were performed using ImageJ software, version 1.54.

The crystallinity parameters were examined using a Shimadzu LabX XRD-6000 (Shimadzu Corporation, Kyoto, Japan) diffractometer with an incident beam of Cu-Kα radiation at λ = 1.5406 Å. The powders produced by drying magnetic nanoparticle solutions were examined in Bragg–Brentano geometry. The diffractograms were obtained in the 20–80° range of 2θ angle, with a scanning step of 0.02° and a scan speed of 0.5°/min.

Spectral analysis in the infrared domain was accomplished with an FTIR spectrometer, Bruker Vertex 70 model (Bruker Optics Company, Bremen, Germany), on a dried magnetic nanoparticle dispersion in potassium chloride to demonstrate how silver and gold attach at the nanoparticle surface.

A Raman confocal microscope LabRAM HR-800, Horiba, Kyoto, Japan was used to record the Raman spectra of the studied nanoparticle samples.

The Lake Shore VSM 7410 (Lake Shore Cryotronics, Westerville, OH, USA) device was used to investigate the magnetization characteristics of dry suspensions under a magnetic field of up to 20 kOe at room temperature, in order to demonstrate the magnetization capacity and evaluate magnetic properties such as particle magnetic core size and coercive and remanent field.

Zeta potential and hydrodynamic diameter measurements were performed using a DelsaNano C analyzer (Beckman Coulter, Brea, CA, USA) in conjunction with an Auto-titrator DelsaNanoAT module.

### 4.4. Antimicrobial Activity Assay

#### 4.4.1. Diffusimetric Method. Bacteria Inhibition Zones

**Figure 22:**
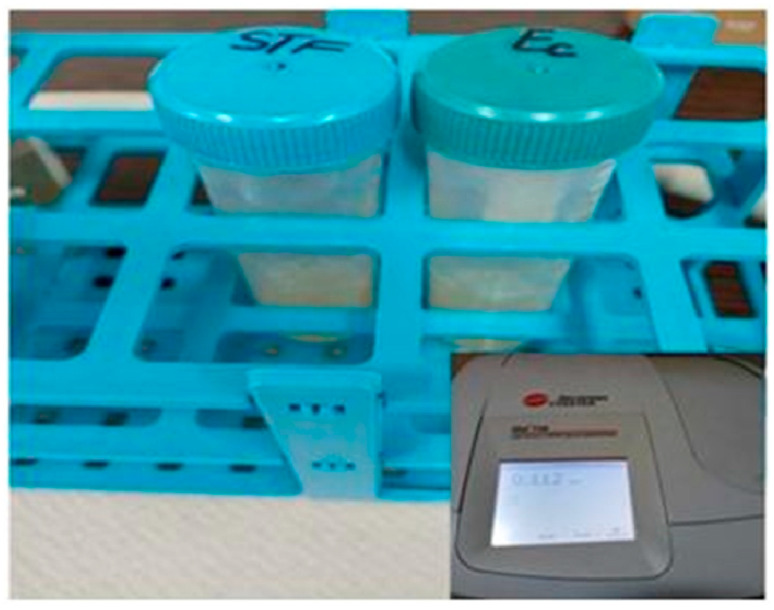
Inoculum standardization.

For microbiological assays, liquid bacterial cultures (18–24 h) were used, from which standardized inocula were prepared, according to the 0.5 McFarland standard (Figure 22).

**Figure 23:**
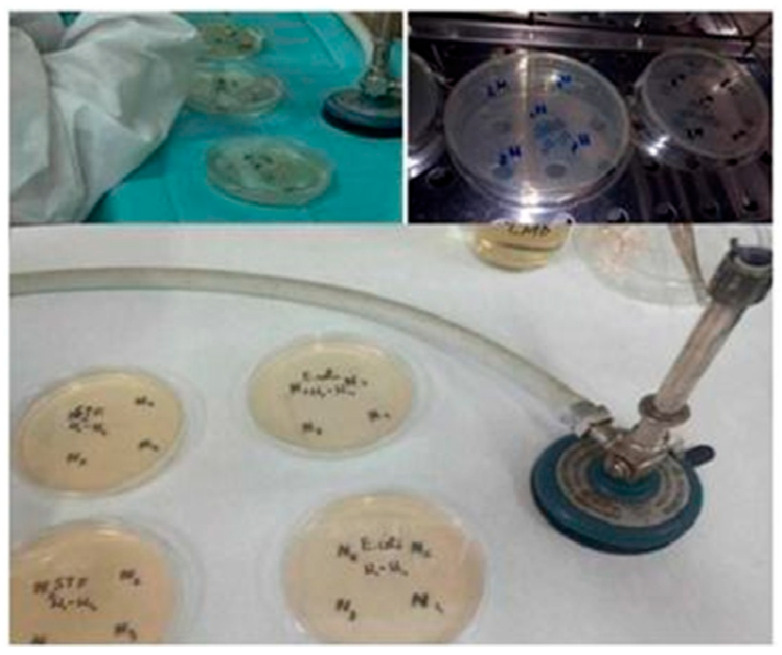
Steps of the diffusimetric method.

They were prepared by dilution in Mueller–Hinton Broth (MHB) culture medium, their turbidity was measured spectrophotometrically at a wavelength of 625 nm in 1 cm cuvettes, the absorbance values were between 0.08 and 0.1. The inoculum standardization was carried out in accordance with the Clinical and Laboratory Standards Institute (CLSI) from 2024. The Kirby–Bauer method was applied using the Mueller–Hinton Agar (MHA) culture medium, from which 30 mL was distributed in each Petri dish, marking the plates and inoculating them with 3 mL of standardized inoculums (Figure 23).

After inoculation and a 10 min rest period, the inoculum was removed, and after another 10 min of rest, four sterile filter paper disks (6 mm diameter) impregnated with 10 μL of each magnetic nanoparticle suspension were placed in each Petri dish, the plates were then placed in a thermostat for 24 h at 37 °C. The diameters of the bacterial inhibition growth zones were measured with a transparent ruler from the center of the paper disks.

#### 4.4.2. The Assay of Minimum Inhibitory Concentration (MIC)

**Figure 24:**
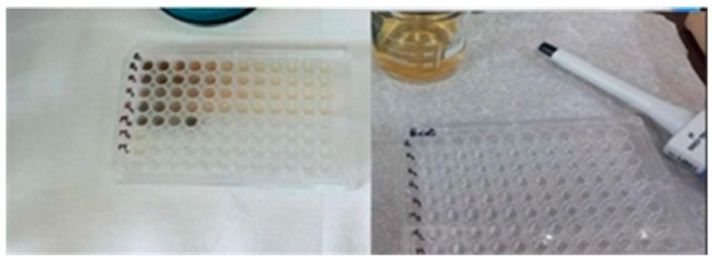
Steps of the method for determining the MIC (minimum inhibitory concentration).

The minimum inhibitory concentration was found by a microdilution technique in nutrient broth culture medium, according to the CLSI standards. Finally, 200 μL of the solution was kept in each well (Figure 24).

Thus, 100 μL of MHB culture medium and 100 μL of magnetic nanoparticle suspension were added to each well of 96-well microdilution plates and, after homogenization, successive double dilutions from 2 to 2048 were added too, along with 100 μL of standardized inoculum, diluted to a concentration of 5 × 10^5^ CFU/mL (CFU—colony-forming units).

The procedure was performed according to the CLSI recommendations [75]; the incubation time was 20 h at a temperature of 37 °C, except for the dilution of the nanoparticles, which were used undiluted beforehand.

Three controls were used to validate the samples: the positive bacterial culture control consisting of 100 μL of the standardized inoculum and 100 μL of culture medium, the nanoparticle control containing 100 μL of magnetic nanoparticles and 100 μL of culture medium, and the negative control was represented by 200 μL of culture medium.

#### 4.4.3. Minimum Bactericidal Concentration Determination (MBC)

The determination of the minimum bactericidal concentration (MBC) is a quantitative test method that is used as a complementary analysis to the determination of the minimum inhibitory concentration (MIC), with the aim of establishing the lowest concentration of a substance to eradicate the bacterial population. The method used to determine the minimum bactericidal concentration of magnetic nanoparticles was adapted based on the CLSI standards [75].

**Figure 25:**
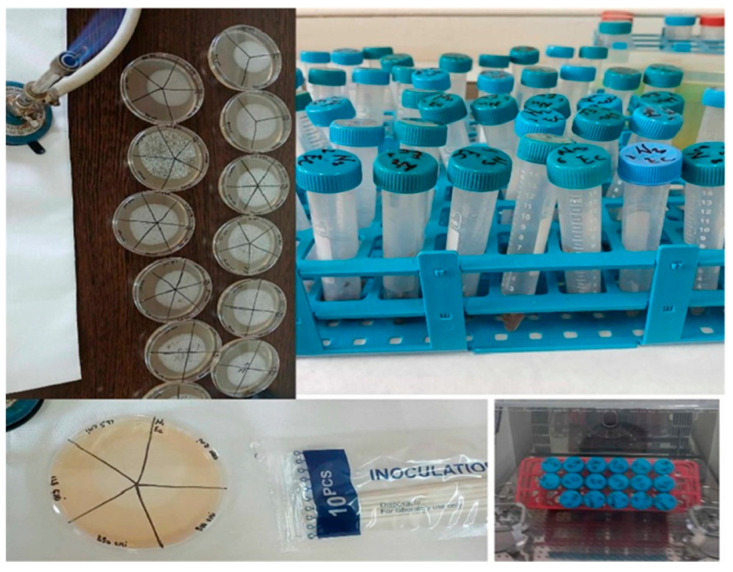
Steps of the method used for the MBC (minimum bactericidal concentration) assay.

Therefore, the minimum bactericidal concentration of magnetic nanoparticles against the reference strains *S. aureus* and *E. coli* was determined based on the minimum inhibitory concentrations. Thus, 10 µL of each sample without visible turbidity, indicating complete inhibition of bacterial growth in the minimum inhibitory concentration stage, were seeded under aseptic conditions in Petri dishes containing 30 mL of sterile Muller–Hinton Agar culture medium to evaluate the ability of nanoparticles of interest to completely eradicate bacterial cells and confirm the bactericidal or bacteriostatic nature of the tested concentrations (Figure 25).

The plates were subsequently incubated for 24 h at 37 °C to reveal the presence/absence of bacterial growth.

## 5. Conclusions

This study reports the successful synthesis of magnetoplasmonic Fe_3_O_4_@Au and Fe_3_O_4_@Ag nanocomposites that formed stable colloidal suspensions and exhibited superparamagnetic behavior and well-defined plasmonic signatures, as confirmed by UV–Vis spectroscopy and dark-field plasmon imaging. These hybrid nanostructures combine magnetic responsiveness and optical functionality, enabling dual-mode operation. Antibacterial evaluation showed that Fe_3_O_4_@Ag possessed the strongest activity, displaying low MIC and MBC values against both *Escherichia coli* and *Staphylococcus aureus*. The higher sensitivity of *E. coli*, consistent with the literature trends regarding Ag–bacteria interactions, highlights the relevance of these nanocomposites for applications requiring broad-spectrum antimicrobial efficiency. The integration of plasmonic and magnetic features offers clear advantages for biomedical applications. Magnetic guidance enables localized delivery and reduces systemic exposure, whereas plasmonic properties provide opportunities for optical monitoring and potential photothermal activation. Their synergistic optical and magnetic properties open pathways for targeted antimicrobial therapies, magnetically guided disinfection, and multimodal diagnostic–therapeutic strategies, laying the groundwork for future translational research in magnetically assisted nanomedicine.

## Figures and Tables

**Table 1:** The mean physical diameter of the studied nanoparticles/nanocomposites, d_TEM_.

Sample	Fe_3_O_4_	Fe_3_O_4_@C_6_H_12_O_6_	Fe_3_O_4_@Ag	Fe_3_O_4_@Au
d_TEM_ (nm)	18.2	20.02	21.1	23.1

**Table 2:** The identified diffraction peaks of the studied samples.

(hkl)	(220)	(311)	(400)	(422)
Fe_3_O_4_@Ag, Fe_3_O_4_ (2θ, °)	30.38	35.70	43.41	54.22
	(511)	(440)	(533)	
	57.41	63.05	74.55	
Ag (2θ, °)	(111)	(200)	(220)	(311)
	38.20	44.40	64.68	77.72
Fe_3_O_4_@Au, Fe_3_O_4_ (2θ, °)	(220)	(311)	(400)	
	30.3	35.64	43.26	
	(511)	(440)		
	57.38	62.72		
Au (2θ, °)	(111)	(200)	(220)	(311)
	38.35	44.76	64.64	77.8

**Table 3:** The magnetization parameters of the studied samples.

Sample	d_M_ (nm)	M_S_ (emu/g)	H_C_ (Oe)	Mr (emu/g)
Fe_3_O_4_	13.1	61.8	20.12	4.3
Fe_3_O_4_@C_6_H_12_O_6_	12	55.13	30.429	2.19
Fe_3_O_4_@Ag	9.6	13.46	23.807	0.63
Fe_3_O_4_@Au	7.3	3.55	74.815	0.42

**Table 4:** Summary on the hydrodynamic diameter, Zeta potential, and polydispersity index.

Sample	D_h_ (nm)/int	PDI Polydispersity Index	Zeta Potential (mV)
Fe_3_O_4_	343	0.274	−15.7
Fe_3_O_4_@C_6_H_12_O_6_	974	0.946	41.84
Fe_3_O_4_@Ag	1193	0.313	23.05
Fe_3_O_4_@Au	579	0.551	−40.57

**Table 5:** The absorption bands in the infrared domain recorded for the studied samples.

Fe_3_O_4_		Fe_3_O_4_@C_6_H_12_O_6_		Fe_3_O_4_@Ag		Fe_3_O_4_@Au	
Band (cm^−1^)	Vibration	Band (cm^−1^)	Vibration	Band (cm^−1^)	Vibration	Band (cm^−1^)	Vibration
632	Fe-O	-	Fe-O	-	Fe-O	-	Fe-O
1075	Fe-O	1055	C-O, C-C	1070	C-O, C-C	1070	C-O, C-C
-	-	1385	C-O-C, C-O-H	1385	C-O-C, C-O-H	1409	C-O-C, C-O-H
1640	O-H	1628	O-H	1640	O-H	1598	O-H
-	-	2890	C-H	2900	C-H	2941	C-H
3446	O-H	3455	O-H	3876	O-H	3384	O-H

**Table 6:** Minimum inhibitory concentration (MIC) values for *Escherichia coli* and *Staphylococcus aureus*.

**Escherichia coli** **ATCC 25922**				**Staphylococcus aureus** **ATCC 25923**		
Magnetic Nanocomposites	Replicate	MIC (mg/mL)	Dilution Factor	Replicate	MIC (mg/mL)	Dilution Factor
Fe_3_O_4_	1	0.187	32	1	0.75	8
	2	0.375	16	2	0.375	16
	3	0.375	16	3	0.375	16
Fe_3_O_4_@C_6_H_12_O_6_	1	0.554	32	1	2.187	8
	2	1.109	16	2	1.109	16
	3	1.109	16	3	1.109	16
Fe_3_O_4_@Ag	1	0.020	512	1	0.020	512
	2	0.005	2048	2	0.020	512
	3	0.010	1024	3	0.010	1024
Fe_3_O_4_@Au	1	3.645	8	1	3.645	8
	2	1.822	16	2	1.822	16
	3	0.911	32	3	1.822	16

**Table 7:** Determination of bacterial population development (MBC).

Tested Microorganisms	Magnetic Nanoparticles and Nanocomposites			
	Fe_3_O_4_	Fe_3_O_4_@C_6_H_12_O_6_	Fe_3_O_4_@Ag	Fe_3_O_4_@Au
*Staphylococcus aureus*	>0.5 mg/mL	>1.468 mg/mL	0.055 mg/mL	>2.429 mg/mL
*Escherichia coli*	>0.312 mg/mL	>0.924 mg/mL	0.027 mg/mL	>2.126 mg/mL

**Table 8:** Reagents needed for the magnetoplasmonic nanostructure preparation.

Nanoparticles	FeCl_3_ × 6H_2_O (134 mM)	FeSO_4_ × 7H_2_O (67 mM)	NaOH (1.7 M)	Glucose (0.1 M/15.62 mM)	HAuCl_4_ (1%)	AgNO_3_ (66.2 mM)
Fe_3_O_4_	3.62 g (100 mL)	1.86 g (100 mL)	3.4 g (50 mL)	−	−	−
Fe_3_O_4_@C_6_H_12_O_6_	3.62 g (100 mL)	1.86 g (100 mL)	3.4 g (50 mL)	3.08 g (171 mL)	−	−
Fe_3_O_4_@Au	3.62 g (100 mL)	1.86 g (100 mL)	3.4 g (50 mL)	4.16 g (231 mL)	9 mL	−
Fe_3_O_4_@Ag	3.62 g (100 mL)	1.86 g (100 mL)	3.4 g (50 mL)	0.056 g (10 mL)	−	0.1125 g 10 mL

## Data Availability

The original contributions presented in this study are included in the article. Further inquiries can be directed to the corresponding authors.

## Abbreviations

The following abbreviations are used in this manuscript:

LSPR	Localized Surface Plasmon Resonance
SEM	Scanning Electron Microscopy
TEM	Transmission Electron Microscopy
EDS	Energy Dispersive X-ray Spectroscopy
SAED	Energy Dispersive X-ray Spectroscopy
XRD	X-ray Diffractometry
VSM	Vibrating Sample Magnetometry
DLS	Dynamic Light Scattering
PDI	Polydispersity Index
FTIR	Fourier Transform Infrared Spectroscopy
